# A pilot study of visceral fat and its association with adipokines, stool calprotectin and symptoms in patients with diverticulosis

**DOI:** 10.1371/journal.pone.0216528

**Published:** 2019-05-08

**Authors:** Kathryn A. Murray, Caroline L. Hoad, Jill Garratt, Mehri Kaviani, Luca Marciani, Jan K. Smith, Britta Siegmund, Penny A. Gowland, David J. Humes, Robin C. Spiller

**Affiliations:** 1 Sir Peter Mansfield Imaging Centre, School of Physics and Astronomy, University of Nottingham, Nottingham, United Kingdom; 2 Nottingham Digestive Diseases Centre and National Institute for Health Research (NIHR) Nottingham Biomedical Research Centre, Nottingham University Hospitals NHS Trust and University of Nottingham, Nottingham, United Kingdom; 3 Trinity Medical Sciences University, Ratho Mill, Kingstown, St. Vincent, West Indies; 4 Gastroenterology, Rheumatology, Infectious Diseases, Charité –Universitätsmedizin, Berlin, Germany; University Hospital Llandough, UNITED KINGDOM

## Abstract

**Background:**

Complications of diverticular disease are increasingly common, possibly linked to increasing obesity. Visceral fat could contribute to the development of symptomatic diverticular disease through its pro-inflammatory effects.

**Objective:**

The study had 2 aims. A) to develop a semi-automated algorithm to measure abdominal adipose tissue from 2-echo magnetic resonance imaging (MRI) data; B) to use this to determine if visceral fat was associated with bowel symptoms and inflammatory markers in patients with symptomatic and asymptomatic diverticular disease.

**Design:**

An observational study measuring visceral fat using MRI together with serum adiponectin, leptin, stool calprotectin and patient-reported somatisation and bowel habit.

**Setting:**

Medical and imaging research centres of a university hospital.

**Participants:**

MRI scans were performed on 55 patients after an overnight fast measuring abdominal subcutaneous and visceral adipose tissue volumes together with small bowel water content (SBWC). Blood and stool samples were collected and patients kept a 2 week stool diary and completed a somatisation questionnaire.

**Main outcome measures:**

Difference in the volume of visceral fat between symptomatic and asymptomatic patients.

**Results:**

There were no significant differences in visceral (*p* = 0.98) or subcutaneous adipose (*p* = 0.60) tissue between symptomatic and asymptomatic patients. However measured fat volumes were associated with serum adipokines. Adiponectin showed an inverse correlation with visceral adipose tissue (VAT) (Spearman ρ = -0.5, *p* = 0.0003), which correlated negatively with SBWC (ρ = -0.3, p = 0.05). Leptin correlated positively with subcutaneous adipose tissue (ρ = 0.8, *p* < 0.0001). Overweight patients (BMI > 25 kgm^-2^) showed a moderate correlation between calprotectin and VAT (ρ = 0.3, *p* = 0.05). Somatization scores were significantly higher in symptomatic patients (*p* < 0.0003).

**Conclusions:**

Increasing visceral fat is associated with lower serum adiponectin and increased faecal calprotectin suggesting a pro-inflammatory effect which may predispose to the development of complications of diverticulosis.

## Introduction

Diverticulosis is a common feature of ageing in the developed world with an incidence which increases with each decade, reaching >50% by the eighth decade and 60% in the ninth [1]. Most patients have asymptomatic diverticulosis, but around 20% develop symptomatic diverticular disease [2]. Studies indicate that hospital admissions for diverticular disease, as well as the incidence of complications and operative interventions are increasing [1, 3, 4], creating a growing disease burden in terms of cost, morbidity and mortality [5].

Early reports of post-mortem examinations [6] suggested 50% of colons from diverticular disease patients had excess fatty tissue compared to 25% of control samples. More recently, long term cohort studies have identified obesity as one of the strongest risk factors for developing symptomatic diverticular disease. According to a Swedish study over a 28-year follow-up period [7], BMI ≥ 30 kg m^-2^ increased the relative risk of of being hospitalised with symptomatic diverticular disease by 4.4. Another study of male health care professionals over an 18 year follow-up period [8] identified that subjects with a BMI ≥ 30 kg m^-2^ had a significantly increased relative risk (RR) of developing diverticulitis (RR = 1.8) or diverticular bleeding (RR = 3.2) compared to those with BMI < 21 kg m^-2^. Obesity has been linked to an increased risk of perforated diverticular disease compared to controls using data from the UK General Practice Research Database (GPRD), RR = 1.4 [9].

While BMI gives a rough measure of total body fat, it is becoming increasingly apparent that visceral fat is much more metabolically active than subcutaneous fat and also a more important predictor of metabolic and intestinal complications such as diverticulitis [10] and Crohn’s disease [2]. Macrophage infiltration into adipose tissue is known to accompany obesity and such macrophages show signs of activation. This leads to the development of an inflammatory state [11], that is shown to decrease when weight is lost. While the link between macrophages in adipose tissue and the morbidities associated with obesity is unclear, several authors have hypothesised that it is specifically macrophage infiltration into intra-abdominal but not subcutaneous fat [12] that is associated with the metabolic syndrome [13, 14]. It has been further hypothesized that visceral fat is not just a storage organ but an important endocrine organ which plays a key role in metabolism [15] leading to metabolic disorders by the release of pro-inflammatory adipokines and cytokines such as adiponectin and leptin. Leptin is pro-inflammatory, stimulating monocytes and macrophages to secrete pro-inflammatory cytokines, nitric oxide and prostaglandins while adiponectin seems mainly anti-inflammatory [16]. The specific role of mesenteric fat in the development of symptomatic colonic diverticular disease has not however yet been explored.

With the increased interest in quantifying abdominal fat, magnetic resonance imaging (MRI) has become the modality of choice for many research studies as it is a non-ionising, multi-planar imaging technique, allowing repeated measurements of adipose tissue distribution across the abdomen [17]. However there is no consensus as to the best method of image acquisition or image segmentation, with many different pulse sequences and image processing algorithms suggested across the literature [17–27]. Semi-automatic and automatic algorithms have been proposed to reduce the observer measurement time and hence make more global estimates of VAT and subcutaneous adipose tissue (SAT) possible.

We have recently developed MRI techniques to non-invasively study the small bowel [28] and colon in health and disease [29]. We have shown a reduction in small bowel water content and accelerated transit in irritable bowel syndrome patients with diarrhoea (IBS-D) compared to healthy controls, both fasting and postprandially [30]. This reduction may reflect increased tone of the small bowel wall. Whether similar effects could be present in symptomatic diverticular patients in whom the predominant bowel pattern is now recognised to be diarrhoea [31] and whether adipokines can exacerbate these symptoms remains to be demonstrated.

This project had two aims; firstly to develop a semi-automated algorithm to measure abdominal subcutaneous adipose tissue (SAT), visceral adipose tissue (VAT) and total adipose tissue (TAT) from 2-echo MRI data. Our second aim was to use this algorithm to investigate the correlations between visceral and subcutaneous adipose tissue and adipokines in symptomatic and asymptomatic diverticular disease and also to assess small bowel water to determine if it was linked to bowel pattern in these patients.

## Materials and methods

The protocol (S1 Protocol) was reviewed and approved by Derby Research Ethics Committee (approval number 10/H0405/80). The study was carried out according to Good Clinical Practice and registered on Clinicaltrials.gov identifier NCT02278770. All patients gave written informed consent.

### Study participants

**Fig 1:**
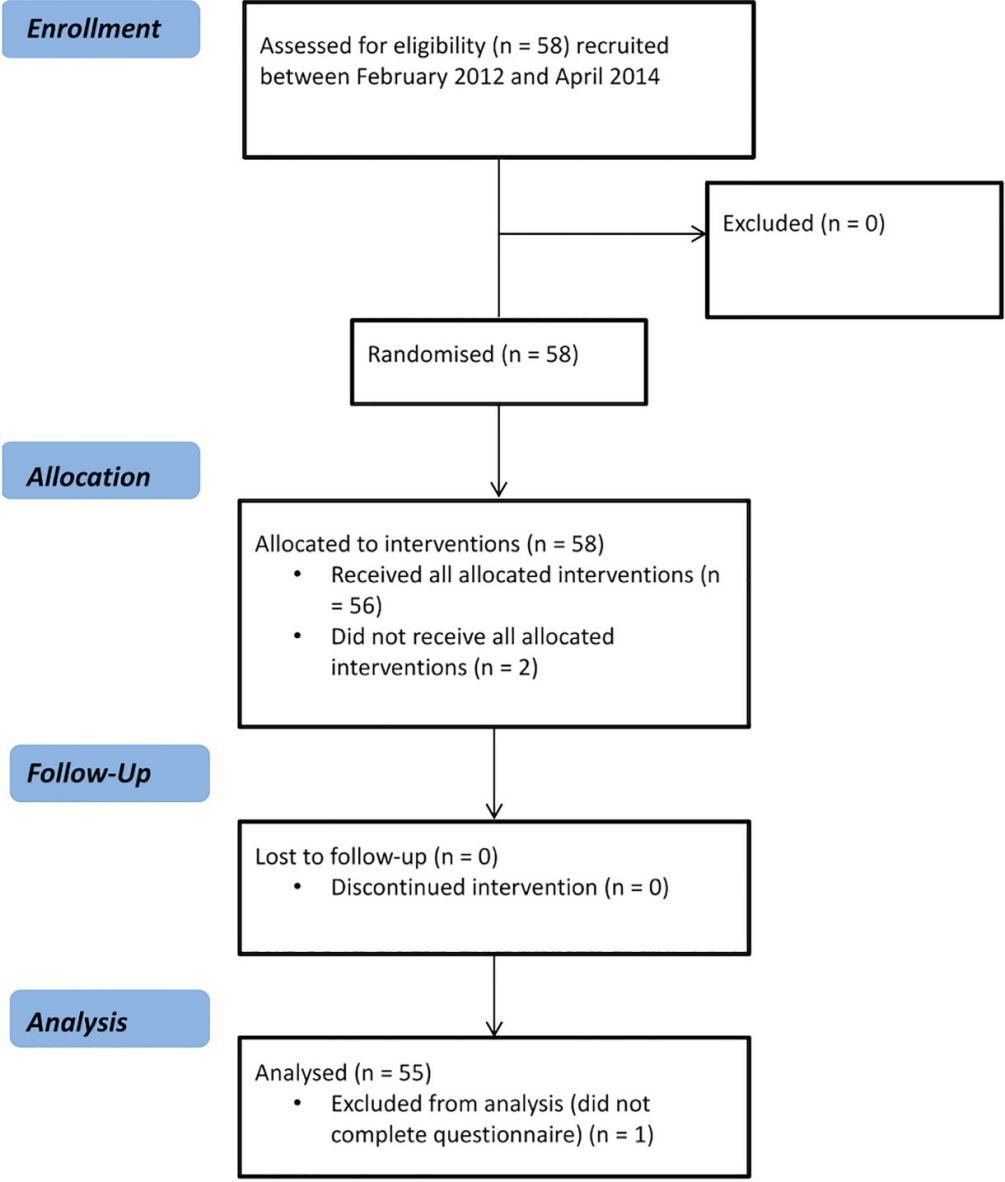
CONSORT flow diagram for the study. The CONSORT checklist can be found in S1 Checklist in the Supporting Information.

58 patients were recruited from gastrointestinal medicine and surgery clinics in Nottingham. Two were not scanned and one failed to complete the 2 week bowel questionnaire leaving 55 patients (24 male; aged 63 ± 10 years with body mass index (BMI) 29.3 ± 5.4 kg m^-2^) who completed the study (Fig 1). Eligibility criteria included patients with a diagnosis for diverticular disease confirmed on endoscopy, barium enema or CT scan and no other gastrointestinal disorders, aged between 18 and 85 years old. Patients were consecutively recruited without any additional selection criteria apart from excluding those on long term non-steroidal anti-inflammatory agents (NSAIDs). Patients were considered symptomatic if they reported lower abdominal pain on 3 or more days/month for more than 3 months, a cut-off paralleling the threshold set by the Rome III Committee for the frequency of symptoms to meet criteria for Irritable Bowel Syndrome[32]. This threshold excludes 94% of the normal population. We felt that this was a reasonable distinction between symptomatic and asymptomatic patients [33]. The presence of multiple somatic symptoms were assessed using Patient Health Questionnaire 12-Somatic Symptom (PHQ12-SS) score, previously shown to be elevated in patients with symptomatic diverticular disease [34]. Scores ≥ 7 were considered high. The Hospital Anxiety and Depression Scale (HADS) questionnaire was also administered at screening in an effort to assess the contribution of anxiety and depression which are known to influence self-reported symptoms[35]. All patients were further screened with a MRI safety screening questionnaire. Out of the 55 patients who completed the study, 10 (6 male, mean age 65 yrs, range 53–73 yrs) were selected based on BMI by someone blind to whether they were symptomatic or asymptomatic, to provide a wide range of BMIs (23–38 kg m^-2^) to facilitate the development of the semi-automated algorithm.

### Study design

Patients were required to make one visit to the study centre, prior to which they were asked to complete a 14-day diary recording the presence of pain, daily bowel habit and Bristol Stool Form score for each bowel movement. They also collected a frozen or freshly passed stool sample for analysis of faecal calprotectin, which they brought to the study centre on the day of their MRI visit. They were asked to cease taking laxatives for 24 hours and antispasmodics for 12 hours prior to scanning. Participants attended the study centre after a 12 hour overnight fast. On arrival, blood samples were taken to assess leptin and adiponectin levels as well as baseline pulse, blood pressure, height and weight measurements. They were then scanned over a period of 10 minutes which completed the study.

#### Magnetic resonance imaging (MRI) protocol

Patients were scanned using a 1.5T, whole-body scanner (Achieva, Philips Medical System, The Netherlands). All participants were positioned supine in the scanner with a 16 element SENSE (sensitivity encoding) receive torso coil around the abdomen. The volumes of subcutaneous and visceral fat were measured using a 3D T1 weighted mDIXON (Modified DIXON) protocol [36] which acquired in-phase, out-of-phase, water only and fat only images in the transverse plane. This acquired 150 slices in 3 stacks; each 50 slice stack acquired in a 13 second breath hold (flip angle = 15^o^, SENSE = 2.0, TE_1_ = 1.8 ms, TE_2_ = 4 ms, TR = 5.4 ms, acquired resolution 1.5 x 1.95 x 6 mm^3^, reconstructed resolution 1.25 x 1.25 x 3 mm^3^). The volume of freely mobile water in the small bowel (small bowel water content, SBWC) was measured using a single short turbo spin echo sequence, which acquired 24 slices with a single 24 second expiration breath hold (TR = 8000 ms, TE = 320 ms, 512 x 512 reconstructed matrix, voxel size 0.78 x 0.78 x 7 mm^3^). This method has been previously described and validated [28].

#### Laboratory analyses

Faecal calprotectin was analysed using a commercially available ELISA kit (Bühlmann Laboratories AG, Schönenbuch, Switzerland). Adiponectin (sBiosciences, Frankfurt, Germany) and leptin (R&D Systems, MN, USA) in blood samples were both analysed by ELISA, according to manufacturers’ protocols.

#### Data analysis

Volumes of abdominal fat were determined using a semi-automatic algorithm written in IDL 6.4 (Research Systems, Boulder Co, USA) (described fully in S1 Methods Supporting Information, which includes all validation studies of the algorithm). The programme uses prior positional knowledge without training data to segment visceral (VAT), subcutaneous (SAT), total adipose tissue (TAT) and abdominal volume in MRI data. The operator first defines the L4/L5 intervertebral disc and selects 25 slices (each slice 3 mm thick) above and below this disc to form a 51-slice region of interest. This region forms the total body mask, and provides volume data for the abdominal volume. A threshold level is set for the fat-only data to generate a fat mask within the total body mask, which was used to differentiate between VAT and SAT. Final volumes of VAT, SAT and abdomen were calculated from the data from the central 30 slices only. As part of the validation process of the algorithm, a subset of 10 datasets were used to compare the fat regions defined by the semi-automatic algorithm with the ‘gold standard’ manual segmentation. SBWC was measured using previously described methods [28].

#### Power and statistical analysis

No previous studies have used MRI measures of VAT and SAT to assess its relationship to symptoms of diverticular disease. However, based on results of Liou who used abdominal MRI to detect fat distribution and found mean abdominal fat volume of 3829 (1106) [37], the sample size required to detect a 30% difference in VAT between groups with a power of 80% and *p* < 0.05 is 16 participants per group. We recruited 17 asymptomatic subjects but recruited a larger number of symptomatic patients to improve our power to detect associations between the different variables assessed.

Statistical analyses were carried out using Prism 6 (GraphPad Software Inc., San Diego, CA). Comparisons between groups were performed using 2 –tailed unpaired *t*-tests and correlations were tested using Spearman’s ρ. Normally distributed data were reported as mean (SD) while median (IQR) was used to report non-normally distributed data.

For the algorithm validation study, a Bland-Altman analysis was used to determine the Bias and 95% Confidence Intervals between the manual and semi-automatic fat volumes. The dice coefficient was used to determine the accuracy of each of the regions defined.

## Results

Of the 55 patients consented, 17 had no pain (labelled asymptomatic) and 38 experienced chronic abdominal pain (labelled symptomatic). There were also 7 participants who recorded an attack of diverticulitis in the preceding year. Clinical details are provided in Table 1.

**Table 1:** Characteristics of symptomatic and asymptomatic patients with diverticulosis^a^.

	Symptomatic	Asymptomatic	*P*^b^
**Age (years)**	64 (54–70)	66 (57–69)	0.7
**Gender**			
% Female	60.5	47.1	
% Male	39.5	52.9	
**BMI (kg m**^**-2**^**)**	29.0 (4.7)	28.3 (4.9)	0.7
**Number of hours/day with pain (hours)**	1.2 (0.6–2.9)	0	0.0001
**Stool frequency (bowel movements/week)**	1.7 (1.3–2.1)	1.6 (1.4–2.4)	1.0
**PHQ-12 SS**	9.0 (6.8–11.0)	4.0 (2.0–7.5)	0.0003
**HAD score**			
Anxiety	6.0 ± 4.2	4.2 ± 3.3	0.14
Depression	3.5 (1.8–6.0)	2.0 (1.0–3.5)	0.09
**Laxative use**	2	2	
**Diverticulitis in the last year**	6	1	0.42

^a^ Normally distributed data are shown as mean (SD) while median (IQR) is used for non-normally distributed data

^b^ *P* values are calculated using unpaired *t* tests for normally distributed data and Mann-Whitney tests when non-normal

29 of the 38 symptomatic patients had an abnormally elevated somatization score (PHQ12-SS) ≥ 7. The somatization score (PHQ-12 SS) was associated with both the number of days with pain (Spearman ρ = 0.6, P < 0.0001) and number of hours / day with pain (Spearman ρ = 0.5, P <0.0003). Increasing BMI was also associated with loose stools (Spearman ρ = 0.3, P = 0.02).

### MRI results

**Fig 2:**
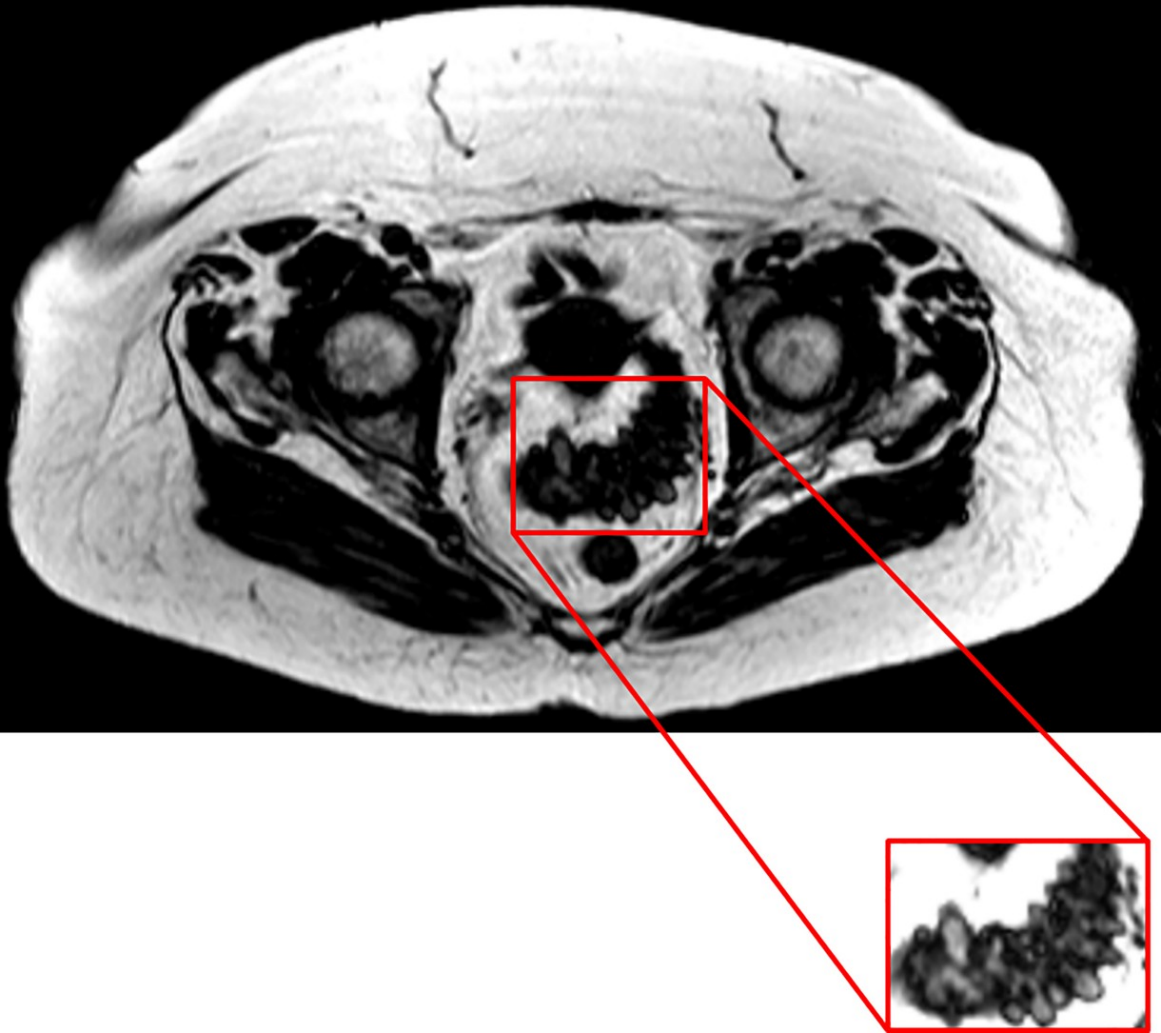
Fat only mDIXON image of the abdominal region of a patient with diverticular disease. The portion of the colon with the diverticula is highlighted in the boxed image. The image was collected using a T1 weighted, water suppressed, sequence where adipose tissue appears bright and allows for its quantification. The colon and other organs appear darkly coloured, providing a contrast for visualisation of the diverticula.

All 55 patients tolerated the non-invasive procedure well and visceral and subcutaneous fat were easily identified using the mDIXON protocol. Diverticula could also be seen predominantly in the sigmoid region, with some visible in the descending colon (Fig 2).

#### Fat measurements

As expected, BMI showed a strong, significant correlation with VAT (Spearman ρ = 0.7, P < 0.0001), SAT (Spearman ρ = 0.7, P < 0.0001) and TAT (Spearman ρ = 0.9, P < 0.0001). BMI also correlated strongly with the abdominal volume of patients (Spearman ρ = 0.9, P < 0.0001). There were however no significant differences between asymptomatic and symptomatic patients with respect to BMI (mean difference [95% CI] 0.5 [-2.3–3.4] kg m^-2^ P = 0.70), nor median volumes of VAT (median difference [95% CI] 39 [-347.5–319.2] mL, P = 0.98), SAT (median difference [95% CI] 67.4 [-391.2–718.9] mL, P = 0.60) and TAT (median difference [95% CI] 422.9 [-586.1–900.1] mL, P = 0.82).

### Adiponectin, leptin and faecal calprotectin

Neither adiponectin nor leptin or calprotectin were significantly different between symptomatic and asymptomatic patients (Table 2). However, patients with calprotectin higher than 50 μg g^-1^ had a lower concentration of plasma adiponectin (median difference [95% CI] 1.45 [0.19–2.69] μg g^-1^, P = 0.02), and a higher volume of VAT, median difference [95% CI] 251.4 [-16.6–626.7] mL, P = 0.06 compared to those with normal calprotectin. BMI (P = 0.45), abdominal volumes (P = 0.26) and leptin concentrations (P = 0.43) were not significantly different. Calprotectin levels of the 7 patients with an attack of diverticulitis in the previous year were not significantly different from those with no such history (median (IQR) 49 (34–193) μg g^-1^).

**Table 2:** Adipokines in symptomatic and asymptomatic patients. Data are shown as median (IQR).

	Symptomatic	Asymptomatic	P
**Adiponectin (**μ**g ml**^**-1**^**)**	4.2 (3.0–6.8)	3.9 (2.5–6.2)	0.8
**Leptin (ng mL**^**-1**^**)**	14.6 (5.9–32.5)	10.79 (4.2–18.9)	0.2
**Calprotectin (**μ**g ml**^**-1**^**)**	44.6 (26.7–116.3)	46.3 (23.6–68.2)	0.6

#### Correlations between adipokines, calprotectin and visceral and subcutaneous fat

**Fig 3:**
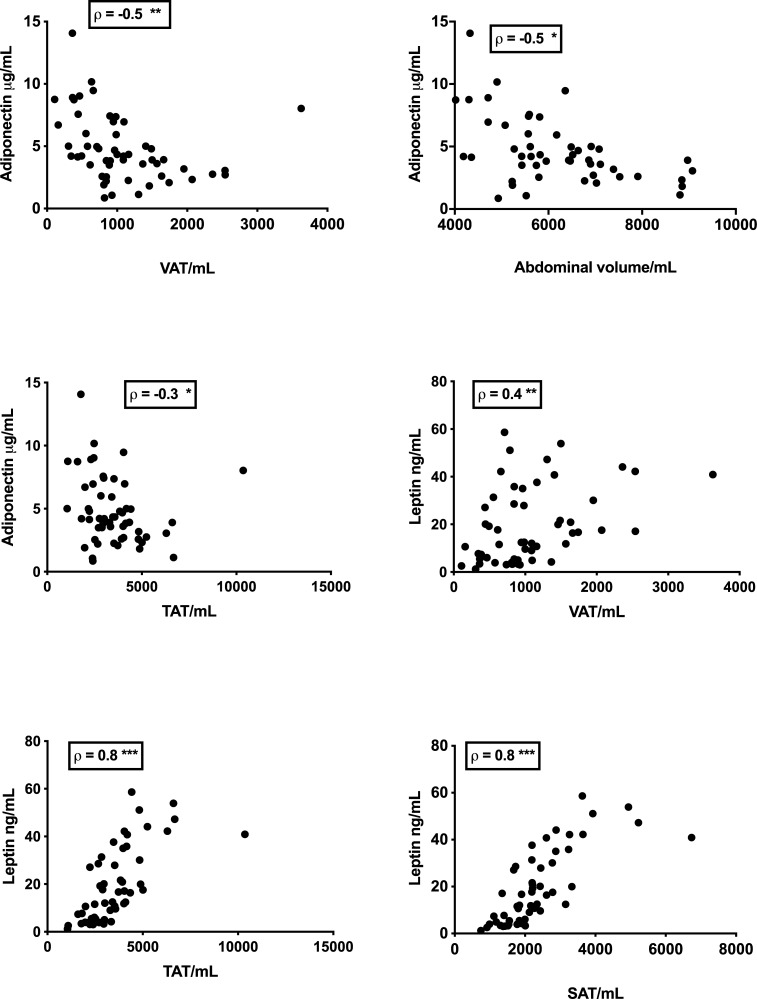
Correlations between adipokines and measures of adipose tissue. Some of the stronger correlations between leptin, adiponectin and MRI measures of fat volume. * = P <0.05, ** = P <0.005, *** = P <0.001.

Correlations between adipokines, calprotectin and adipose tissue are shown in Fig 3. This figure shows strong negative correlations between adiponectin and fat, while leptin is shown to correlate strongly with SAT and TAT, but less strongly with VAT.

The median faecal calprotectin concentration was 46.3 (27–100) μg g^-1^, 46% being above the upper limit of normal in our laboratory of 50 μg g^-1^. While there were no correlations between calprotectin and fat for the full dataset of patients, for the overweight subgroup a post-hoc analysis showed there were modest correlations between calprotectin and VAT (Spearman ρ = 0.3, P = 0.05), BMI (Spearman ρ = 0.3, P = 0.04) and abdominal volume (Spearman ρ = 0.4, P = 0.01).

#### Correlation of objective markers with bowel habit and pain frequency

Average stool form ranged from 1.9–6 and this measure was shown to correlate positively with BMI and negatively with adiponectin (Table 3). BMI also correlated positively with the number of days with loose stool and negatively with adiponectin. Calprotectin correlated negatively with adiponectin. All other correlations are shown in Table 3.

**Table 3:** Association between bowel symptoms and objective measures of adiposity, adipokines and calprotectin.

		Spearman ρ	*P*
**Average Bristol Stool Form Score**			
	BMI	0.4	0.005
	Adiponectin	-0.3	0.0095
	VAT	0.3	0.03
**Number of days/ week hard stool**			
	VAT	-0.3	0.03
	VAT/SAT	-0.4	0.004
	Adiponectin	0.5	0.0002
**Adiponectin**			
	BMI	-0.4	0.0008
	Calprotectin	-0.4	0.009

#### Small bowel water content (SBWC)

Fasting SBWC was not significantly different between symptomatic and asymptomatic patients being mean (SD) 72.0 (79.5) mL and 66.7 (40.5) mL respectively. SBWC was however found to correlate negatively with VAT (Spearman ρ = -0.3, P = 0.05), SAT (Spearman ρ = -0.4, P = 0.006, TAT (Spearman ρ = -0.4, P = 0.003), leptin (Spearman ρ = -0.4, P = 0.002) and BMI (Spearman ρ = -0.3, P = 0.04). There were no significant differences between patients with BMI < 25 kgm^-2^ and those with BMI ≥ 25 kgm^-2^ mean difference 7 (26), 95% CI [–46–60] mL, P = 0.40.

#### Validation of the fat segmentation algorithm

Comparison of the volumes from 10 diverticular disease patients measured using the semi-automatic algorithm and manual segmentation are given in Table 4. A broad range of volumes were measured across the subjects for the different adipose tissue regions. The dice coefficients for all the different regions were greater than 0.95, showing excellent agreement between the boundaries defined by the algorithm and those defined by the ‘gold standard’ manual segmentation.

**Table 4:** Comparison of manual and semi-automatic definitions of adipose tissue regions (N = 10) obtained across 30 slices.

	VOLUMES (ml)			RATIOS	
	VAT	SAT	TAT	VAT/SAT	VAT/TAT
**Mean Semi-Auto Data**	1020±310	2540±1180	3660±1350	0.46±0.19	0.29±0.08
**Range**	620–1490	1000–5230	2030–6690	0.20–0.85	0.16–0.42
**Mean Manual Data**	1020±320	2540±1170	3660±1350	0.46±0.19	0.29±0.08
**Range**	600–1500	1000–5230	2030–6690	0.20–0.86	0.16–0.42
**B-A Bias (MAN-AUTO)**	-0.2	-27.8	0.0	0.004	-0.001
**B-A 95%CI**	-25.2 to 24.8	-62.4 to 6.8	-0.2 to 0.2	-0.008to 0.016	-0.009 to 0.007
**Mean Dice Co-efficient**	0.983±0.009	0.994±0.002	1.000±0.000	N/A	N/A

## Discussion

In this study of adults with diverticular disease, we developed a semi-automated algorithm to measure abdominal adipose tissue from MRI data and used it to determine if visceral adipose tissue was associated with bowel symptoms and inflammatory markers in patients with symptomatic and asymptomatic diverticular disease. There were no significant differences in adipose tissue volume between symptomatic and asymptomatic patients, but there were some associations noted between adipose tissue volumes, stool calprotectin and serum adipokines.

We report here that in overweight diverticular disease subjects, increasing VAT is associated with an increase in gut inflammation as assessed by faecal calprotectin. Faecal calprotectin; a S100A 8/9 heterodimer found in the cytosol of neutrophils and macrophages [38], is widely used to assess gut inflammation. Our data is further evidence that increasing visceral fat is pro-inflammatory and may play a role in the association between obesity and the complications of diverticular disease. A recent report examining visceral fat obtained from patients undergoing gastric bypass surgery for morbid obesity, has shown that calprotectin is generated by activated macrophages in visceral adipose tissue, possibly under the influence of TNF alpha [39]. Its correlation with visceral obesity in our overweight patients supports the idea that increased VAT may enhance the inflammatory response in diverticular disease which could predispose to bacterial translocation and microabscess formation or visceral hypersensitivity.

We have previously shown that these are linked to increased mucosal expression of a range of pro-inflammatory mediators including TNFɑ, IL6, monocyte chemotactic peptide 1 (MCP1) and prostaglandin E (PGE) in diverticular disease [40]. Others have also reported a link between faecal calprotectin, mucosal lymphocyte count and symptoms in diverticular disease, implying low grade inflammation could cause pain and bowel disturbance [41]. One of the strongest predictors of the development of pain in diverticulosis is prior diverticulitis. During this inflammation, the gut barrier is breached and bacteria enter the visceral fat, which in experimental colitis can be shown to act as second layer of defence [42]. This inflammatory state may well persist even after the original mucosal injury has healed and could contribute to the increased risk of symptomatic diverticular disease associated with obesity [43]. The association between calprotectin and VAT could also be indirect since a high fat diet is associated both with obesity and increased gut permeability [44]. It should be noted that we did not restrict the patients diet as the only clinical trials of diet have failed to show that red meat alters faecal calprotectin [45].

Adiponectin was found to correlate negatively with VAT, TAT, abdominal volume, BMI and calprotectin. This is in keeping with past studies, where concentration of adiponectin decreases in obesity but increases when weight is lost [46]. Indeed, adiponectin release from the omentum of women undergoing hysterectomy has been negatively correlated with the amount of visceral fat, while release from SAT was not associated with any metabolic abnormalities [47]. The negative correlation of adiponectin with calprotectin is in keeping with the anti-inflammatory nature of adiponectin, reduction of which in obesity, may contribute to a pro-inflammatory state.

Unlike adiponectin, concentrations of leptin were very strongly correlated with SAT, TAT and abdominal volume, while correlations with VAT were only modest. Leptin mRNA is expressed at a greater rate in SAT than it is in VAT, possibly due to the differences in VAT and SAT cell sizes [48], and this is in keeping with what was seen for our patients. Leptin was also inversely correlated with SBWC, which in turn was inversely correlated with SAT, TAT and BMI.

This finding of a negative correlation between SBWC and adiposity together with the link between BMI and loose stools is in keeping with epidemiological data which links diarrhoea with increased BMI [49] and our own data, showing reduced small bowel water in irritable bowel syndrome patients with diarrhoea [50]. The underlying mechanism is unclear and requires further study, but leptin has been reported to increase transit by altering the sensitivity to CCK, which accelerates distal small intestinal transit [51]. Faster transit could lead to the reduced water in the small bowel we have observed.

We found increased VAT, which represents the more metabolically active fat, was linked to frequent soft stools. This agrees with Lee et al., [52] who report that visceral obesity is associated with an increased risk of IBS-D.

Recent reports have highlighted the link between obesity and complications of diverticular disease [4]. Obesity is clearly linked to the risk of type II diabetes and the metabolic syndrome, thought to be mediated via the production of pro-inflammatory cytokines by visceral fat. Our study attempted to determine possible mechanisms underlying the epidemiological data linking obesity with inflammatory complications of diverticular disease. We confirmed, using our improved imaging methods, that serum levels of leptin are closely correlated with total body fat, and that this correlation is greater than for VAT which only accounts for a fraction of total body fat. In contrast serum adiponectin, an anti-inflammatory adipokine derived from visceral fat, correlates more strongly negatively with more metabolically active VAT than with total abdominal fat.

Our method is patient acceptable though limited by patient size, a study weakness which is being corrected by the large bore scanners now becoming available which make scanning very obese patients feasible. Future work should focus on the effect of weight loss on markers of gut inflammation which would show whether the relationship we have observed is causal or a spurious association mediated by diet or changes in microbiota.

This should be regarded as a pilot study since the numbers were small and only included a few patients with previous diverticulitis. It is clinically important to distinguish between recurrent acute diverticulitis (where the pain comes in episodes lasting several days separated by long periods of no symptoms) and IBS-like diverticular disease where the pain is often daily and short lived. A future study comparing larger numbers of both groups is needed to clarify whether visceral fat is linked to symptoms in either of these 2 groups.

One of the strengths of this study was the use of MRI which allowed the collection of multiple slices through the abdomen [53], which has been shown to improve the accuracy of visceral adipose tissue measurements. Our semi-automated programme analysed a larger cross sectional area centred at the L4/L5 intervertebral disc without the use of large amounts of training data or complicated statistical models suggested by other previous published techniques [22, 25]. Our MRI method overcomes the differences in fat storage between men and women which makes other single slice methods unreliable [54]. MRI methods are also superior to CT scanning, as there is no harmful radiation, and multiple slices can be collected in just a few breath holds. While the link between calprotectin and VAT was a post hoc discovery and though plausible mechanistically, needs repeating to ensure it is not due to chance, the method shows promise and can be applied to other diseases such as Crohn’s, where the effects of abdominal adiposity can be investigated.

## Supporting information

S1 Checklist S1_Checklist. (DOC)

S1 Protocol S1_Protocol. (DOC)

S1 Methods S1_Methods. (DOCX)
