# Supplementary material for: A pilot study of visceral fat and its association with adipokines, stool calprotectin and symptoms in patients with diverticulosis
Source: PLoS One. 2019 May 8;14(5):e0216528. doi: 10.1371/journal.pone.0216528 (PMC6505945; doi:10.1371/journal.pone.0216528)
Supplement: S1 Protocol — (DOC) [file pone.0216528.s002.doc]

**S1 Protocol**


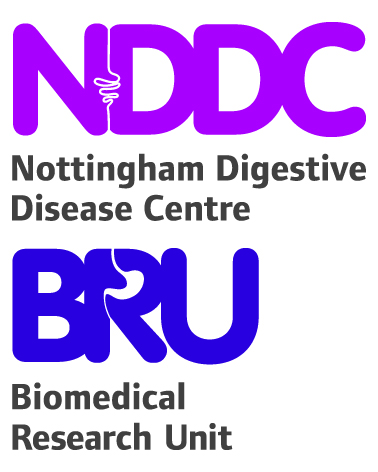

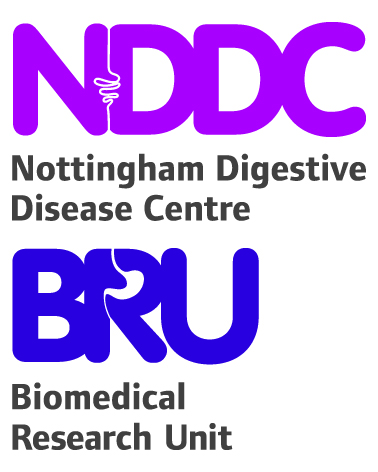

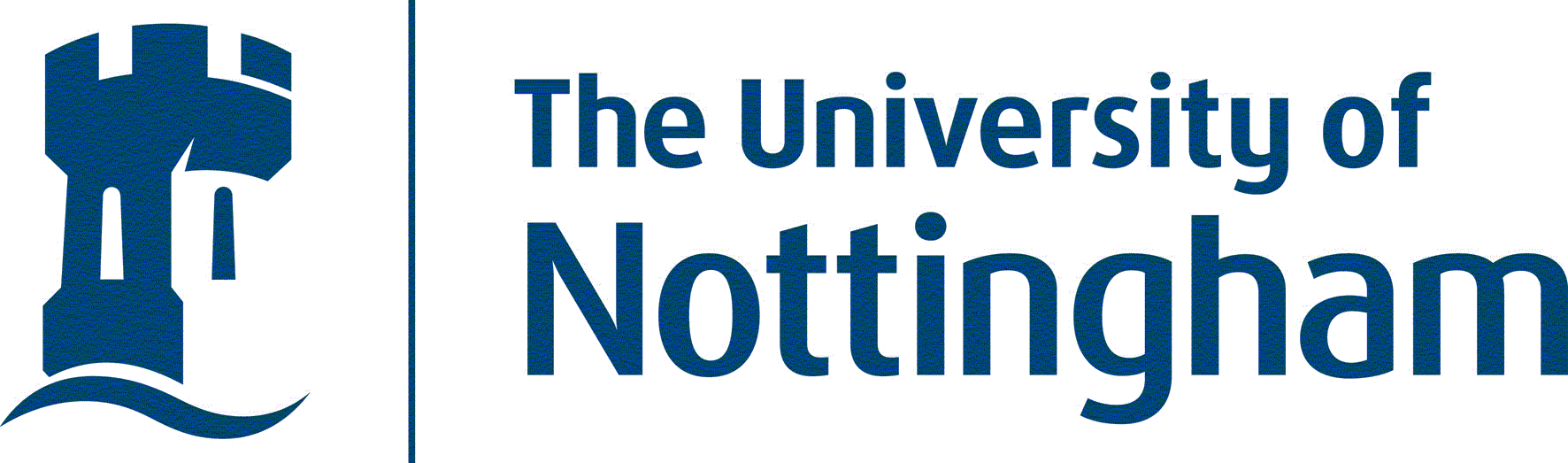


**Pilot study: MRI study of Diverticular disease symptoms**

**and its relationship to visceral adipose tissue**.

**Final Version 1.0**

**1.7.10**

**Short title:** *DD Obesity MRI study*

**Acronym:** *DDOMRI*

**REC reference:** *insert when allocated*

**Trial Sponsor:** University of Nottingham

**Funding Source:**

*Funding sources are being sought*

STUDY PERSONNEL AND CONTACT DETAILS

**Sponsor:** University of Nottingham

Contact name Mr Paul Cartledge

Head of Research Grants and Contracts

Research Innovation Services

King’s Meadow Campus

Lenton Lane

Nottingham

NG7 2NR

**Chief investigator:**

Prof RC Spiller

Professor of Gastroenterology

Nottingham Digestive Disease Centre

E Floor, West Block,

Nottingham University Hospitals,

Nottingham,

NG7 2UH

Tel: 0115 8231032

Email: [robin.spiller@nottingham.ac.uk](mailto:robin.spiller@nottingham.ac.uk)

**Co-investigators:**

Miss JK Smith

Academic Clinical Fellow,

Department of Surgery,

E Floor, West Block,

Nottingham University Hospitals,

Nottingham,

NG7 2UH

Tel: 0115 82 31170

jan.smith@nottingham.uk

Mr DJ Humes Dr L Marciani,

Lecturer in Surgery, RCUK Fellow

Department of Surgery, Nottingham Digestive Disease Centre,

E Floor, West Block, E Floor, West Block,

Nottingham University Hospitals, Nottingham University Hospitals,

Nottingham, Nottingham

NG7 2UH NG7 2RD

Tel: 0115 82 3 Tel: 0115 9518276.

[david.humes@nottingham.ac.uk](mailto:david.humes@nottingham.ac.uk) [luca.marciani@nottingham.ac.uk](mailto:luca.marciani@nottingham.ac.uk)

Prof. P Gowland Miss Kay Head

Professor of Physics Research Assistant

Sir Peter Mansfield MRI Centre, Sir Peter Mansfield MRI Centre

School of Physics and Astronomy, School of Physics and Astronomy

Nottingham University, Nottingham University,

University Park, Nottingham University Park, Nottingham

NG7 2RD NG7 2RD

Tel: 0115 951 4754

[penny.gowland@nottingham.ac.uk](mailto:penny.gowland@nottingham.ac.uk) kay.head@nottingham.ac.uk

Dr. S Francis Mrs D Bush,

Lecturer in Physics Specialist Research Nurses,

Sir Peter Mansfield MRI Centre, Department of Surgery,

School of Physics and Astronomy, E Floor, West Block,

Nottingham University, Nottingham University Hospitals

University Park, Nottingham Nottingham,

NG7 2RD NG7 2UH

Tel: 0115 846 6518

[susan.francis@nottingham.ac.uk](mailto:susan.francis@nottingham.ac.uk) Debbie.bush@nottingham.ac.uk

**Study Coordinating Centre:**

Nottingham Digestive Disease Centre BRU

E Floor, West Block,

Queen’s Medical Centre

Nottingham University Hospitals,

Nottingham,

NG7 2UH

SYNOPSIS

| Title | Pilot study: MRI study of Diverticular disease symptoms and its relationship to visceral adipose tissue. |
| --- | --- |
| Acronym | DDOMRI |
| Short title | DD obesity MRI study |
| Chief Investigator | Prof RC Spiller |
| Objectives | To determine differences in adipose deposition and adipokines on diverticular disease symptoms and small bowel water using MRI in participants. |
| Study Configuration | Single centre parallel groups |
| Setting | Secondary care |
| Sample size estimate | No previous studies have used MRI measures of visceral and subcutaneous fat to assess its relationship to symptoms in diverticular disease.  However based on results using abdominal MRI to detect fat distribution by Liou et al in 2006[50], the sample size required to detect a 30% difference in visceral fat between groups with a power of 90% and p<0.05 is 35 participants per group. |
| Number of participants | 105  Expected 500 participants needed to contacted to identify 105 participants for MRI part of study |
| Eligibility criteria | 1. Participants must have symptomatic and asymptomatic Diverticular disease confirmed on endoscopy/ telescope test, barium enema or CT scan   (Symptomatic disease is define as lower abdominal pain >1hr on 3 or more days per month for longer than 3 months.)   1. 18 – 85 years of age. 2. Signed informed consent |
| Description of interventions | Participants will include;   - 1. 35 participants with symptomatic DD and a patient heath questionnaire 15 score of >7   2. 35 participants with symptomatic DD and a patient heath questionnaire 15 score of <7   3. 35 participants with asymptomatic DD   Participants will complete one of each of the following on one occasion: Bowel symptom questionnaires, bowel habit diary, patient heath questionnaire 15, Hospital and Anxiety Depression score questionnaire, McGill pain questionnaire.  Participants will provide 1 stool sample and have a MRI scan of their abdomen and give 20ml of blood from a peripheral vein. |
| Duration of study | 14 days  Recruitment planned for January 2011  Duration of study up to 3 years |
|  |  |
| Outcome measures | Primary End-point  1. Difference in the volume of visceral fat compared to abdominal subcutaneous fat between symptomatic and asymptomatic DD groups  Secondary End-Points. Differences between:   1. Adiponectin and leptin serum levels between symptomatic and asymptomatic DD groups 2. The incidence of diarrhoea between participants with a BMI <25Kgm^-2^ and those >25Kgm^-2^. 3. Adiponectin and leptin serum levels between DD groups with and without diarrhoea 4. Small and large bowel water and lumen diameter in symptomatic and asymptomatic DD groups 5. Calprotectin levels between symptomatic and asymptomatic DD groups. 6. Microbiological gut profile between symptomatic and asymptomatic DD groups. |
| Statistical methods | Statistical analysis will carried out with the use of Prism 4 (GraphPad Software Inc, San Diego, CA). Comparisons between groups were performed by using 2-tailed unpaired t test or the Mann–Whitney's U test, for normal and non-normal data respectively. |

# ABBREVIATIONS

|  |  |
| --- | --- |
| AE | Adverse Event |
| BRU | Biomedical Research Unit |
| CI | Chief Investigator overall |
|  |  |
| CRF | Case Report Form |
| DD | Diverticular Disease |
| DAP | Data Analysis Plan |
| DMC | Data Monitoring Committee |
|  |  |
|  |  |
| GCP | Good Clinical Practice |
| HDL | High density lipoprotein |
|  |  |
| IBS | Irritable Bowel Syndrome |
| ICF | Informed Consent Form |
| IL | Interleukin |
|  |  |
| NDDC | Nottingham Digestive Disease Centre |
| NHS | National Health Service |
|  |  |
| P/GIS | Parent / Guardian Information Sheet |
| PI | Principal Investigator at a local centre |
| PIS | Participant Information Sheet |
|  |  |
| REC | Research Ethics Committee |
| R&D | Research and Development department |
|  |  |
|  |  |
|  |  |
|  |  |
|  |  |
|  |  |
|  |  |
|  |  |

**TABLE OF CONTENTS**

[STUDY PERSONNEL AND CONTACT DETAILS 2](#__RefHeading___Toc271804836)

[Primary End-point 5](#__RefHeading___Toc271804837)

[Secondary End-Points. 5](#__RefHeading___Toc271804838)

[ABBREVIATIONS 7](#__RefHeading___Toc271804839)

[STUDY BACKGROUND INFORMATION AND RATIONALE 10](#__RefHeading___Toc271804840)

[Diverticular disease 10](#__RefHeading___Toc271804841)

[Altered bowel habit and diverticular disease 14](#__RefHeading___Toc271804842)

[Causes of alteration in bowel habit in diverticular disease 15](#__RefHeading___Toc271804843)

[Alteration of small bowel water in IBS 15](#__RefHeading___Toc271804844)

[STUDY OBJECTIVES AND PURPOSE 17](#__RefHeading___Toc271804845)

[Aim 17](#__RefHeading___Toc271804846)

[PURPOSE 17](#__RefHeading___Toc271804847)

[Hypotheses 17](#__RefHeading___Toc271804848)

[PRIMARY OBJECTIVE 17](#__RefHeading___Toc271804849)

[SECONDARY OBJECTIVES 17](#__RefHeading___Toc271804850)

[STUDY DESIGN 18](#__RefHeading___Toc271804851)

[STUDY CONFIGURATION 18](#__RefHeading___Toc271804852)

[Methods 18](#__RefHeading___Toc271804853)

[Participant groups 18](#__RefHeading___Toc271804854)

[MRI study day protocol 18](#__RefHeading___Toc271804855)

[Biological samples 19](#__RefHeading___Toc271804856)

[Stool samples 19](#__RefHeading___Toc271804857)

[Data Analysis 19](#__RefHeading___Toc271804858)

[STUDY MANAGEMENT 19](#__RefHeading___Toc271804859)

[DURATION OF THE STUDY AND PARTICIPANT INVOLVEMENT 20](#__RefHeading___Toc271804860)

[End of the Study 20](#__RefHeading___Toc271804861)

[SELECTION AND WITHDRAWAL OF PARTICIPANTS 20](#__RefHeading___Toc271804862)

[Recruitment 20](#__RefHeading___Toc271804863)

[Participant Selection and Recruitment 20](#__RefHeading___Toc271804864)

[Inclusion criteria 22](#__RefHeading___Toc271804865)

[Exclusion criteria 22](#__RefHeading___Toc271804866)

[Expected duration of participant participation 23](#__RefHeading___Toc271804867)

[Participant Withdrawal 23](#__RefHeading___Toc271804868)

[Informed consent 23](#__RefHeading___Toc271804869)

[STUDY REGIMEN 24](#__RefHeading___Toc271804870)

[Compliance 25](#__RefHeading___Toc271804871)

[Potential adverse events 25](#__RefHeading___Toc271804872)

[TRANSPORT AND STORAGE OF THE TISSUES AND LABORATORY ANALYSES 25](#__RefHeading___Toc271804873)

[STATISTICS 27](#__RefHeading___Toc271804874)

[Methods 27](#__RefHeading___Toc271804875)

[Sample size and justification 27](#__RefHeading___Toc271804876)

[ETHICAL AND REGULATORY ASPECTS 27](#__RefHeading___Toc271804877)

[ETHICS COMMITTEE AND REGULATORY APPROVALS 27](#__RefHeading___Toc271804878)

[INFORMED CONSENT AND PARTICIPANT INFORMATION 28](#__RefHeading___Toc271804879)

[RECORDS 28](#__RefHeading___Toc271804880)

[Study Forms 28](#__RefHeading___Toc271804881)

[Source documents 29](#__RefHeading___Toc271804882)

[Direct access to source data / documents 29](#__RefHeading___Toc271804883)

[*RECORD RETENTION AND ARCHIVING* 30](#__RefHeading___Toc271804884)

[DATA PROTECTION 30](#__RefHeading___Toc271804885)

[QUALITY ASSURANCE & AUDIT 30](#__RefHeading___Toc271804886)

[INSURANCE AND INDEMNITY 30](#__RefHeading___Toc271804887)

[STUDY CONDUCT 30](#__RefHeading___Toc271804888)

[STUDY DATA 31](#__RefHeading___Toc271804889)

[RECORD RETENTION AND ARCHIVING 31](#__RefHeading___Toc271804890)

[DISCONTINUATION OF THE TRIAL BY THE SPONSOR 31](#__RefHeading___Toc271804891)

[STATEMENT OF CONFIDENTIALITY 31](#__RefHeading___Toc271804892)

[PUBLICATION AND DISSEMINATION POLICY 32](#__RefHeading___Toc271804893)

[USER AND PUBLIC INVOLVEMENT 32](#__RefHeading___Toc271804894)

[STUDY FINANCES 32](#__RefHeading___Toc271804895)

[Funding source 32](#__RefHeading___Toc271804896)

[Participant stipends and payments 32](#__RefHeading___Toc271804897)

[SIGNATURE PAGES 33](#__RefHeading___Toc271804898)

[REFERENCES 34](#__RefHeading___Toc271804899)

# STUDY BACKGROUND INFORMATION AND RATIONALE

## Diverticular disease

Colonic diverticulosis is the most common structural abnormality of the colon, yet our understanding of it is rudimentary. It affects 5% of people in their 5^th^ decade and up to 66% of the elderly population in the United Kingdom[1]. It is responsible for substantial morbidity with 68,000 hospital admissions recorded per year in the UK and it contributes to about 2,000 deaths.

The definitions of diverticulosis and diverticular disease were established by the European Association for Endoscopic Surgery consensus development meeting in 1999[2]:

“Colonic diverticular disease is a condition seen mostly in the sigmoid region. It is characterized structurally by mucosal herniation through the colonic wall, generally accompanied by muscular thickening, elastosis of the taenia coli, and mucosal folding. This condition may be asymptomatic (*diverticulosis*) or associated with “symptoms,” termed *diverticular disease,* which may be complicated or uncomplicated. The term *diverticulitis* is used to indicate superadded inflammation involving the bowel wall. Other pathologic complications include perforation, fistula, obstruction, and bleeding.”

Studies using national databases of hospital admissions suggest its incidence and/or complications are increasing. A recent study from the United States reported a 26% increase in admission for acute diverticulitis between 1998 and 2005. The rise in admission rates were greatest in younger patients e.g. 45-64 years and 18-44 years[3].

**Obesity and Gastrointestinal disease**

Increasing evidence suggests a link between obesity and gastrointestinal dysfunction and disease. Abdominal pain, bloating and diarrhoea have been correlated with a BMI >25kg/m^2^ from population based studies[4-7]. Decreased colonic compliance and pain sensation has also been associated with a BMI >25Kg/m^2^ [8]. Other supportive evidence includes a noticed decrease in the incidence of reported gastrointestinal symptoms with increased exercise and healthy diets (high fruit and fibre)[9]. However, different eating patterns between obese and lean individuals does not fully explain the effect of BMI on reported gastrointestinal symptoms[10].

In a recent scintigraphic gastrointestinal transit study of 287 IBS patients (118 C-IBS, 139 D-IBS, 30 M-IBS) and 170 healthy volunteers, colonic filling and transit were measured at 6, 8, 24 and 48hrs. The researchers found that transit measured at 8, 24 and 48hrs showed statistical difference, especially those with a BMI >30Kg/m^2^ when adjusted for age, gender and subject types[11]. The reasons underlying the association between obesity and altered colonic transit are not known, but may include:

1. Overwhelming of the jejunal absorption by a high carbohydrate and fat loaded diet, resulting in a higher osmotic chyme being passed distally, with retention of water content.
2. Increased bile acid excretion leading to cholerrheic diarrhoea as a high BMI has been associated with a greater incidence of idiopathic bile acid malabsorption compared to normal weight individuals[12].

The influence of adipose tissue on gastrointestinal immunological and hormonal function may also play a role[13, 14].

**Obesity and Diverticular disease**

There is increasing evidence that obesity is linked to complications of diverticular disease. In Parks’ 1968 study of colonic specimen, 50% of DD samples had excess fat attached compared to control samples with no noticeable gender difference[15].

More recent epidemiological studies have also suggested an association. In a 5 year retrospective study of 61 patients with diverticulosis, a significant difference in body mass index (BMI) was demonstrated between those with no symptoms and those who developed perforation or recurrent episodes of diverticulitis[16]. In a larger prospective cohort study, a BMI >30kg/m^2^, high waist circumference and a high waist to hip ratio was shown to significantly increase the relative risk of diverticular bleeding and diverticulitis[17]. Studies of patients under the age of 40yrs with acute diverticulitis have also demonstrated an association with obesity[18]. In addition a correlation between obesity and uncomplicated symptomatic diverticular disease has been found in a Nottingham study[19]

With the prevalence of obesity increasing in westernised populations, the risk of complications from diverticular disease is likely to increase as well. However there is little understanding of how obesity increases diverticular complications.

**Possible mechanisms linking obesity to symptomatic diverticular disease**

1. **Association with lifestyle risk factors**

It has been speculated that the increase in incidence of diverticular disease and its complications[3], may be linked to changing western lifestyle. Western diets, especially the lack of cellulose in the diet, and decreased mobility increase the risk of developing diverticular symptoms[20, 21]. As these are linked with the development of obesity, a high BMI may be a surrogate marker for other lifestyle factors that predispose to diverticular complications. Obesity has also been linked to other gastrointestinal pathologies[22-24], including colorectal carcinoma[24-26], chronic inflammation[25, 27] and changes in gastrointestinal microflora.

1. **Inflammation**

Visceral fat has secretes systemic factors that influences on metabolic, endocrine and immunological functions. Visceral fat can also have local influences upon the organs it surrounds, in a paracrine manner. Adipocytes secrete a variety of important compounds:

1. **Adipokines and cytokines**
   1. **Adiponectin**

Adiponectin is produced from adipocytes [28, 29]. Adiponectin is inversely related to BMI and its levels are greater in women than men[24]. Low adiponectin levels and higher concentrations of cytokines, e.g. TNF alpha, IL6, IL8, IL10 and IL1beta, are thought to be involved in the development of insulin resistance and possibly insulin growth factor(IGF) and IGF binding proteins 1 and 2[24].

Adiponectin also has anti-angiogenic and anti-inflammatory effects. Deficient mice have more severe form of chemically induced colitis that normal mice[30, 31]. Variations in adiponectin concentrations have also been reported in UC and Crohn’s disease (CD). Adiponectin and leptin have been shown to be up-regulated in CD compared to visceral fat samples from colon cancer or diverticular disease patients[32].

- 1. **Leptin**

Leptin is an adipocyte specific hormone. It acts on appetite and bodyweight and circulating levels have been associated with colonic cancer. It has similar effect on oesophageal and colonic cell lines, increasing angiogenesis, cell proliferation and metalloproteinase expression.

Leptin levels correlate with white adipose fat mass and is coded for by the obese (ob) gene, while its receptor is coded by the diabetes (db) gene. Levels are greater in women than men, even when adjusted for by BMI. It has multiple actions in satiety and inflammation (see figures from Lago et al 2007[33]).

It acts on a variety of immunological cells such as monocytes, neutrophiles, basophils, NK cells and T cells. In T cells it effects the activation and balance between TH1 and TH2 cytokine production, causing a pro-inflammatory TH1 response. Leptin is itself increased by mediators of inflammation such as lipopolysaccharide (LPS), IL-1 and IL-6. Knockout mouse models suggest leptin is important in development of inflammatory diseases, such as colitis. However its exact mechanism is not fully understood[33].

Leptin has been identified as a critical component in the development of tactile allodynia in a partial sciatic nerve ligation (PSNL) mouse model of pain. After PSNL, adipocytes around the epineurioum increased expression of leptin. Mice given antibodies against leptin and leptin knockout mice failed to produce allodynia, which was reverse after administration of leptin. It is thought that leptin acts by attracting Macrophages, with leptin receptors to injured nerves and caused production of pro-nociceptive mediators Leptin induces iNOS, COX-2 and MMP-9 via phosphorylated signal transducers and activators of transcription 3 (pSTAT3) pathways, which up-regulate the expression of leptin[34]. Peritoneal administration of leptin has also been found to cause pain in mice models[35].

In clinical studies, individual levels of adipokines from synovial fluid, from arthritic knees undergoing joint replacements, did not correlate well with pain scores. However adioponectin-leptin ratios were significantly correlated with pain scores from the Short form McGillPain questionnaire[36]. Waist circumference, BMI and female sex have also been found to be predictive of leptin levels in synovial fluid[37]. Leptin has also been suggested to play a role in endometriosis patients with chronic pelvic pain[38]. From Lago et al 2007[33].

1. **Inflammatory cells**

Adipose macrophage numbers is associated with degree of an individual’s adiposity. They are attracted from the peripheral circulation by monocyte chemoattractant protein 1 (MCP-1) and TNF alpha or from pre-adipocyte differentiation. These cells are thought to influence cytokine production and insulin resistance. There is speculation that the type of macrophage e.g. type 1 ‘classically activated’ or type 2 ‘alternatively activated’, may also be influenced by obesity. Type 2 macrophages are activated by IL4 or/and 13 and secrete IL-10, suppressing adaptive immunity, and allowing tissue remodelling and promoting tumour development and progression[24].

## Altered bowel habit and diverticular disease

Persistent or intermittent alteration of bowel habit is a common complaint in patients with diverticulosis and diverticular disease. A recent study suggests that 28.5% of patients with diverticulosis have altered bowel habit[39] and 16.5% frequent lower abdominal pain. Diverticular patients often complain of constipation (11.7% functional and 18.4% chronic) and/or diarrhoea (6.4% functional and 12.2% chronic)[39]. This is especially the case in the over 65yrs age group there is a correlation between diarrhoea predominant IBS (OR 1.9 95% CI 1.1 - 3.3 P 0.02) and mixed IBS (OR 3.0 95%CI 1.2 - 7.4 P 0.02) and diverticulosis[39].

## Causes of alteration in bowel habit in diverticular disease

The cause of altered bowel habit in IBS and DD is not well understood. In IBS animal models, electrophysiological and bio-molecular studies suggest a long term interaction of the immune system and the visceral nervous system[40]. Changes in micro-organisms within the gastrointestinal tract may also contribute to the maintenance of an altered immune system and are thought to play a role in symptomatic diverticular disease[41] and post-infectious IBS[42].

Genetic techniques for micro-organism profiling have recently been used in IBS. Differences in flora have been identified in diarrhoea predominant IBS (D-IBS)[43, 44]. Increased prevalence of Proteobacteria and Firmicutes and a reduction of Actinobacteria and Bacteroidetes species have been found in D-IBS samples compared to controls[43]. The interaction between the gastro-intestinal flora, immune system and the visceral nervous system has been implicated in a number of functional and inflammatory gastrointestinal diseases[45]. In DD, Diverticular associated colitis and bacterial overgrowth have also been blamed[46].

Therefore, the causes of constipation, diarrhoea or mixed picture of diverticular disease and IBS are probably multi-factorial and include changes in small bowel and colonic dysmotility, mucosal inflammation and intestinal microflora.

## Alteration of small bowel water in IBS

Small bowel water content has been previously difficult to assess. Seven to eleven litres of fluid passes through the upper digestive system per day. However the majority (80%) is absorbed so that only 2 litres enters the colon[47]. There is a natural balance between the excretion and absorption of fluid within the gastrointestinal system, which if disturbed can lead to diarrhoea or constipation.

Until recently studies required intestinal intubation and perfusion or aspiration of low viscosity liquid ‘meals’ which do not mimic normal physiological states and can influence gastrointestinal function. New MRI techniques have now been developed[48] and have shown a reduction in small bowel water content (SBWC) of fasted D-IBD patients compared to healthy volunteers[49]. This reduction can be mimicked by intravenous injections of corticotrophin releasing factor (CRF), a mediator of the stress response which IBS patients are know to be hypersensitive to. Stress in known to be important in the pain and diarrhoea in IBS and similar effects could be present in diverticular disease where diarrhoea predominates. Stress also activates mucosal mast cells and may contribute to the proinflammatory bias of the mucosal immunocyutes seen in IBS. Whether adipokines can exacerbate this remains to be demonstrated.

# STUDY OBJECTIVES AND PURPOSE

## Aim

1. To determine differences in adipose deposition and adipokines on diverticular disease symptoms and small bowel water using MRI in participants.

## PURPOSE

## Hypotheses

1. DD Patients with a higher proportion of visceral fat will have increased incidence of painful symptoms and loose stool compared to patients with higher subcutaneous fat distribution.
2. Patients with DD symptoms will have decreased levels of adipokines compared non-symptomatic DD controls but have increased levels of circulating pro-inflammatory cytokines.
3. Patients with diarrhoea predominant diverticular disease will show similar MRI results to D-IBS patients, having reduced fasting small bowel water content and a narrower gastrointestinal lumen diameter (contracted small bowel) but increased colonic water.

## PRIMARY OBJECTIVE

Difference in the volume of visceral fat compared to abdominal subcutaneous fat between symptomatic and asymptomatic DD groups

## SECONDARY OBJECTIVES

Differences between:

1. Adiponectin and leptin serum levels between symptomatic and asymptomatic DD groups
2. The incidence of diarrhoea between participants with a BMI <25Kgm^-2^ and those >25Kgm^-2^.
3. Adiponectin and leptin serum levels between DD groups with and without diarrhoea
4. Small and large bowel water and lumen diameter in symptomatic and asymptomatic DD groups
5. Calprotectin levels between symptomatic and asymptomatic DD groups.
6. Microbiological gut profile between symptomatic and asymptomatic DD groups.

# STUDY DESIGN

## STUDY CONFIGURATION

This is a pilot study, single centre, 14 day study comprising of (i) questionnaires of bowel habit, pain and anxiety, a bowel habit and pain 14 day diary, (ii) biological samples: 1 stool sample and 1 * 20ml blood sample and (iii) an MRI scan of the abdomen to identify fat distribution and large and small bowel water content.

# Methods

## Participant groups

We will recruit n = 35 participants in each group (Total 105). Participants will include;

- 1. 35 participants with symptomatic DD and a patient heath questionnaire 15 score of >7
  2. 35 participants with symptomatic DD and a patient heath questionnaire 15 score of <7
  3. 35 participants with asymptomatic DD

The DD groups will be age and sex matched.

Having excluded those with e.g severe co-morbidity, alcoholism or drug dependence or inability to give informed consent (see exclusion criteria), we will invite participation in the MRI study day.

## MRI study day protocol

Participants will be required to make 1 visit to the department. Prior to the visit we will ask participants to complete a 14 day bowel habit diary sheet and provide a stool sample, to bring on the day of their MRI visit.

The participants will visit the ***Brain and Body Centre*** after a overnight fast (fast of 12 hrs food and 6 hr for clear fluids). They will be asked to omit any laxatives for 24hrs and antispasmodics for 12hrs prior to the MRI study day. The participants will be screened and consented for the MRI study. They will have baseline pulse, blood pressure, height and weight, waist and hip circumference measured and recorded and will complete a safety questionnaire to ensure there are no contraindications to MRI (i.e. have metal implants or pacemaker).

Participants will be shown the 1.5T MRI Philips Achieva scanner. They will be positioned supine in the scanner with a parallel imaging body coil wrapped around the abdomen. Several different imaging sequences will used to optimally image the different regions of the gut as follows;

1. SBWC will be assessed with a single shot, fast spin echo sequence. This scan will take approximately 10 minutes.
2. A dual-gradient echo imaging sequence will be used to visualize anatomy. This scan will take approximately 10 minutes.

Including set-up and imaging, the volunteers spend approximately 20 minutes inside the magnet.

## Biological samples

### Stool samples

Participants will be asked to freeze their stool samples in the domestic freezer prior to bringing it to the B&B centre. Pots, gloves, bags and detailed instructions will be provided to the participants to prevent contamination of food by faeculent material. Samples will be brought to the B&B centre by the participant and frozen at -20^o^C so that the sample time of >-20^o^C is kept to a minimum time of 1.5hrs. All samples will be anonymised and stored at -20oC in the Nottingham Digestive Disease Centre freezers. Samples will be analysed for calprotectin and genetic techniques (e.g. quantitative PCR or microarray) to identify diversity of gastrointestinal microflora between groups.

**Blood samples**

20ml of blood will be taken to assess plasma levels of;

Adipokines – adiponectin and leptin

Inflammatory mediators – e.g. TNF alpha, IL-6, IL-10, IL-8, IL-4, IL-1, CRP

Biochemical characteristics – insulin, fasting glucose, triglycerides, liver function tests

All samples will be anonymised and stored at -80^o^C in the Nottingham Digestive Disease Centre freezers.

## Data Analysis

The visceral and subcutaneous fat, and SBWC images will be analyzed with the use of in-house software as previously described and validated by intubation studies at 1.5T[48].

## STUDY MANAGEMENT

The study has been adopted by the Nottingham Digestive Disease Centre (NDDC). The CI will oversee the day to day running of the study. Study supplies will be purchased through the NDDC and shipped to the Brain and Body imaging centre, as required. Miss Jan Smith will be responsible for ordering supplies. Dr L Marcianai will be responsible for co-ordinating analysis of the MRI samples. Miss J Smith will be responsible for co-ordinating analysis of the questionnaires

The data will be collected from participants into individual participant folders. All study documents will be labelled with the participants study code. Files will be stored in a locked filing cabinet in a locked office in the NDDC on E Floor West Block, Queen’s Medical Centre. Prof RC Spiller is the custodian of the study data.

## DURATION OF THE STUDY AND PARTICIPANT INVOLVEMENT

Participants will be required to make 1 visit to the department. Prior to the visit we will ask participants to complete a 14 day bowel habit diary sheet and provide a stool sample, to bring on the day of their MRI visit.

On the day of the visit they will be consented for the scanning part of the study, give a 20 ml blood sample and have a 20-25 minute MRI scan of their abdomen. The visit will last approximately 1 hour.

### End of the Study

The end of the study for the participant is on the MRI scanning day. No further follow up of the participant will be planned. If medical abnormalities are found in the data collected, the participants will be referred to appropriate NHS Heath care professions for continued investigation and treatment. The research team will not formally follow up any participant where a medical abnormality has been detected once appropriate NHS care has been organised.

## SELECTION AND WITHDRAWAL OF PARTICIPANTS

### Recruitment

## Participant Selection and Recruitment

Participants will be recruited from gastrointestinal medicine and surgery clinics and databases of patients with diverticular disease held at the NDDC, who have previously expressed interest and or have previously participated in research. The initial approach will be from a member of the participant’s usual care team or researcher. Information about the study will also be on display in the relevant clinical areas. Potential participants from clinics will be initially approached by a member of their usual care team, which may include the investigator, regarding the study. Participants will only be approached directly by the research team if they have been consented previously to being contacted for future research. NDDC databases will be reviewed by Miss J Smith, Mr D Humes or Mr Govind Jumbu (NDDC Database Manager).

A newspapers advert may also be placed in local newspapers and associated news websites e.g. the Evening post, if required. Interested individuals who want more information about the study will be able to contact the research team using the telephone, email and postal address provided. An answer phone message for the study will be provided to allow messages to be recorded at any time. The research team to return calls to the advert-responders, briefly describe the study to the potential participant, check that they are suitable for the study using a structured proforma and answer any questions before sending out the patient information sheet, introduction letter and questionnaire.

The letters to potential participants contain a reply slip/questionnaire consent form and questionnaire, which they can return to give the research team permission to verify the diagnosis of diverticular disease by checking hospital records or contacting the potential participant’s GP. A Medical trained profession from the research team will review any hospital records in order to verify a diagnosis of diverticular disease. This may be required even for potential participants recruited through clinic as their normal care team may not be able to fully assess the potential participant for the inclusion and exclusion criteria.

The patient’s general practitioner may be contacted by post initially with a standardised letter, copy of the patient’s signed consent form to allow information to be given to the research team and a copy of the patient information leaflet. If no response is received within 2 weeks, a member of the research team will phone the GP practice to confirm they have received the original letter. If not, the letter will be sent again by post or secure fax to the GP practice to be completed by the GP. The completed form can be returned by post to the NDDC or by secure fax. If the completed form is not received with in following 2 weeks, the GP practice will be contacted again to identify any problems. If the GP practice fails to give out the requested information after this further contact, the potential study participant will be informed that without confirmation of their diagnosis of diverticular disease we are unable to include them in the study. If the potential study participant wants to contact their GP themselves directly, the research team will forward a copy of the GP letter with a free post envelope to him/her.

If the research team cannot confirm the diagnosis of diverticular disease from past radiological or endoscopic investigations, we will not recruit the potential participant to the study. All consent and procedures will occur at Nottingham University under Prof Spiller's research team.

The investigator(s) from the research team or a member of the participant’s usual care team, will inform the participant or their nominated representative (other individual or other body with appropriate jurisdiction), of all aspects pertaining to participation in the study. Written information will be provided with an introductory letter, participant information leaflet, questionnaire, reply form and return envelope. If written information cannot be provided at the time of the clinic visit, verbal consent will be gained for the information to be posted to the participant’s home address.

The participants will have up to 2 weeks to decide. If they return the reply slip and/or questionnaire, they will be contacted by a member of the research team regarding a time to attend for the MRI visit or to discuss the study in more detail. This may be via telephone or post. The questionnaire will allow clarification of gastrointestinal specific inclusion and exclusion criteria. Other inclusion and exclusion criteria may be clarified by post, on the telephone or by using patient hospital records, with patient consent. This is to avoid unnecessary travel and inconvenience to the participant if this information has not been previously obtained or is ambiguous from the questionnaire.

### Inclusion criteria

1. Participants must have symptomatic and asymptomatic Diverticular disease confirmed on endoscopy/ *telescope test, barium enema or CT scan*

*(Symptomatic disease is define as lower abdominal pain >1hr on 3 or more days per month for longer than 3 months.)*

1. 18 – 85 years of age.
2. Signed informed consent

### Exclusion criteria

General exclusions

1. Pregnant or lactating women.
2. Severe co-morbidity; e.g. heart failure, respiratory failure, alcoholism or drug dependence,
3. Inability to give informed consent.
4. If the participant has taken part in any other study on campus in the last 3 months they will not be able to take part in this study.
5. Inability to lie supine
6. Inability to stop laxatives for 24hrs and antispasmodics or ondansetron for 8 hours prior to the commencement of the MRI study.
7. Using long-term NSAIDs (non-steroidal anti-inflammatory agents e.g. ibuprofen), antibiotics or immunosuppressant drugs
8. Antibiotics within last 3 months
9. Other gastrointestinal inflammatory problems e.g. ulcerative colitis, Crohn’s or Coeliac disease

MRI exclusions

1. Have a metallic implant e.g.
   1. Cardiac pacemaker or Implanted cardiac defibrillator
   2. Metallic heart valves
   3. Aneurysm clips
   4. Carotid artery vascular clamp
   5. Neurostimulator
   6. Insulin or infusion pump or implanted drug infusion device
   7. Non-removable cochlear, otologic, or ear implant
2. Have shrapnel inside the body
3. Ever had metallic fragments in the eye
4. Claustrophobia

### Expected duration of participant participation

Study participants will be participating in the study for 14 days

### Participant Withdrawal

Participants may be withdrawn from the study either at their own request or at the discretion of the Investigator. It will also be explained that they can withdraw at any time but attempts will be made to avoid this occurrence. The participants will be made aware that this will not affect their future care. In the event of their withdrawal it will be explained that their data collected so far cannot be erased and we will seek consent to use the data in the final analyses where appropriate. Remaining tissue samples will be destroyed if the participant so wishes.

### Informed consent

All participants will provide written informed consent. The Consent Form will be signed and dated by the participant before they enter the study. The Investigator will explain the details of the study and provide a Participant Information Sheet, ensuring that the participant has sufficient time to consider participating or not. The Investigator will answer any questions that the participant has concerning study participation.

Informed consent will be collected from each participant before they undergo any interventions (including physical examination and history taking) related to the study. One copy of this will be kept by the participant, one will be kept by the Investigator, and a third will be retained in the patient’s hospital records.

Should there be any subsequent amendment to the final protocol, which might affect a participant’s participation in the study, continuing consent will be obtained using an amended Consent Form which will be signed by the participant.

If needed, the usual hospital interpreter and translator services will be available to assist with discussion of the trial, the participant information sheets, and consent forms, but the consent forms and information sheets will not be available printed in other languages. It will be explained to the potential participant that that entry into the study is entirely voluntary and that their treatment and care will not be affected by their decision. It will also be explained that they can withdraw at any time but attempts will be made to avoid this occurrence. In the event of their withdrawal it will be explained that their data collected so far cannot be erased and we will seek consent to use the data in the final analyses where appropriate.

## STUDY REGIMEN

Please see diagram below.


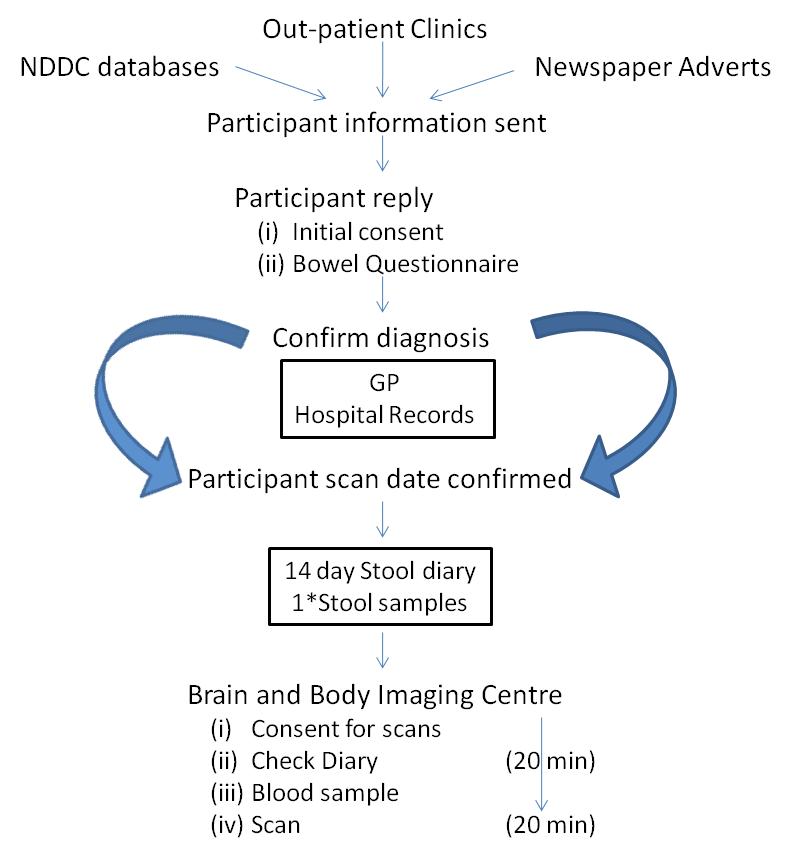


Participants who have completed the questionnaires will be invited to attend the MRI scanning session if they;

1. Are confirmed to have a radiological or endoscopic diagnosis of diverticular disease
2. Meet the inclusion or exclusion criteria

It is expected that up to 500 potential participants will be sent the letter of introduction and questionnaires to obtain 105 participants who meet the study criteria.

Participants will attend the MRI scanning session after a overnight fast (fast of 12 hrs food and 6 hr for clear fluids). They will be asked to omit any laxatives for 24hrs and antispasmodics for 12hrs prior to the MRI study day.At all over times of the study, participants can take their normal medication and eat and drink whenever they like. This request is made as they can interfere with the measurement of small and large bowel water content.

### Compliance

The questionnaires and diary sheets will be assessed at the MRI scanning visit. The participant will be accepted as compliant if they have completed the bowel symptom questionnaire and at least 10 days or more of the diary sheet. Failure to provide a stool sample will not result is exclusion from taking part further in the MRI scanning or blood sampling aspects of the study.

## Potential adverse events

MRI

There is no evidence that MRI scanning affects pregnancy however we will err on the side of safety and exclude pregnant women and those with known contraindications to MRI scanning. These include metal implants, metallic foreign bodies and pace makers. All potential participants will be carefully screened to ensure no MRI exclusion criteria are present. If the participant has a tattoo they will be advised of the potential for heating of the metallic tattoo dyes which may cause a burn to the skin. They will asked to sign a further consent form to acknowledge they have been advise of the potential problems that can occur with tattoos and are happy to proceed with the scan.

Medical research staff will be present to provide appropriate care in the unlikely event any other adverse event occurs, Adverse events will not be routinely reported to the REC, unless the Chief Investigators requests.

All participants will receive a copy of the standardised participant information sheet which details how to contact the investigators should the need arise. The investigators will be the first contact point for adverse events. However, if the investigators are unavailable, or on advise of the research team, the participant will be asked to contact their GP or Accident and Emergency Department.

# TRANSPORT AND STORAGE OF THE TISSUES AND LABORATORY ANALYSES

Blood samples:

Blood samples which will be processed by the Nottingham University Hospitals Biochemistry laboratory will be labelled with the patients name, date of birth, date of sample taken and NUH hospital number as per standard NHS protocol for blood sample labelling. Trial blood samples for cytokines and adipokines with be separated to collect the serum/plasma and frozen as per the standard operating procedure for these samples. All cryo-tubes will be labelled with the patient initials, date of birth, MRI allocated study number and date the sample was taken.

Samples will be frozen at the Brain and Body centre and transported by a member of the research team to the NDDC laboratories where they will be stored in the NDDC -80^o^C freezers until processed. Serum/Plasma samples will be transported to the Nottingham City Hospital laboratories for cytokine and adipokines analysis by a member of the research team using standard procedures as advised by Dr Spendlove’s team at the Nottingham City Hospital. Dr Spendlove’s team will advise and help carry out analysis of the samples.

Participant detail, initials, date of birth, NUH hospital number, MRI number and date of scan will be maintained in a paper version (locked in a filing cabinet in a locked office at the NDDC) and on a password protected Microsoft access database held on the University of Nottingham server. All study documents will be anonymised using the MRI number, initials and date of birth.

Stool Samples

Samples will be collected by the researchers on the MRI study day. The participant will collect 1 stool sample and split it between 3 pots to allow ease of analysis. They will be stored at 4^o^C (on ice) before being transported by a member of the research team to the NDDC laboratory freezers and frozen at -20^o^C. All samples will be labelled with the patient initials, date of birth, MRI allocated study number and date the sample was taken. Stool will be analysed for Calprotectin and/or microbiological organisms. In order to accurately identify microbiological organisms, Bacterial DNA may be extracted from stool samples and analysed.

Participants will be asked if they would like to donate their study samples to as yet unspecified future studies at the end of the study. This is optional for the participants. If they would like to donate any remaining samples, they will be asked to sign the corresponding consent form section. Any remaining samples (with consent) will be retained under the UoN HTA licence reference 12265. If they decline, the samples will be taken for the study, but destroyed at the end of the study following the HTA principles.

# STATISTICS

### Methods

Statistical analysis will carried out with the use of Prism 4 (GraphPad Software Inc, San Diego, CA). Comparisons between groups were performed by using 2-tailed unpaired *t* test or the Mann–Whitney's *U* test, for normal and non-normal data respectively by Dr Luca Marciani and Miss Jan Smith

### Sample size and justification

No previous studies have used MRI measures of visceral and subcutaneous fat to assess its relationship to symptoms in diverticular disease.

However based on results using abdominal MRI to detect fat distribution by Liou et al in 2006[50], the sample size required to detect a 30% difference in visceral fat between groups with a power of 90% and p<0.05 is 35 participants per group.

# ETHICAL AND REGULATORY ASPECTS

The Research Ethics Committee approval will be obtained prior to initiation. Similar studies have been performed previously with no ethical problems. No ethical problems are foreseen in this project however there is a risk that we will notice something unexpected on the abdominal scans, although we are taking these scans for scientific research. They are not the same as scans collected by doctors for medical purposes and the images will not be looked at by a radiologist. All biochemical tests will be analysed by an NHS laboratory.

However if we should notice something abnormal on abdominal imaging or in biochemical analysis of blood samples the principal investigator will arrange for a specialist radiologist or other medical professional to examine the scans or blood tests and advise if any further action that should be taken. The research team and/or a medical doctor will explain the situation to the participant and on any further action that should be taken. If the participant agrees, the principal investigator will also contact the participant’s G.P. (as chosen by the participant in the Consent Form to make them aware of any referral or treatment that may be needed.

The analysis used for cytokines and adipokines does not have relevance to current clinical diagnosis or practice. However, if during the study, new information should result in any of our cytokine or adipokines assessments being utilised in clinical practice, the research team will review our study records, gain advice from specialists and assist in contact any participants who may be affected in order to make them aware of any referral or treatment that may be needed.

## ETHICS COMMITTEE AND REGULATORY APPROVALS

The study will not be initiated before the protocol, consent forms and participant and GP information sheets have received approval / favourable opinion from the Research Ethics Committee (REC), and the respective National Health Service (NHS) Research & Development (R&D) department. Should a protocol amendment be made that requires REC approval, the changes in the protocol will not be instituted until the amendment and revised informed consent forms and participant and GP information sheets (if appropriate) have been reviewed and received approval / favourable opinion from the REC and R&D departments. A protocol amendment intended to eliminate an apparent immediate hazard to participants may be implemented immediately providing that the REC are notified as soon as possible and an approval is requested. Minor protocol amendments only for logistical or administrative changes may be implemented immediately; and the REC will be informed.

The study will be conducted in accordance with the ethical principles that have their origin in the Declaration of Helsinki, 1996; the principles of Good Clinical Practice, and the Department of Health Research Governance Framework for Health and Social care, 2005.

## INFORMED CONSENT AND PARTICIPANT INFORMATION

The process for obtaining participant informed consent or assent and parent / guardian informed consent will be in accordance with the REC guidance, and Good Clinical Practice (GCP) and any other regulatory requirements that might be introduced. The investigator or their nominee and the participant or other legally authorised representative shall both sign and date the Consent Form before the person can participate in the study.

The participant will receive a copy of the signed and dated forms and the original will be retained in the Study records. A second copy will be filed in the participant’s medical notes and a signed and dated note made in the notes that informed consent was obtained for the study.

The decision regarding participation in the study is entirely voluntary. The investigator or their nominee shall emphasize to them that consent regarding study participation may be withdrawn at any time without penalty or affecting the quality or quantity of their future medical care, or loss of benefits to which the participant is otherwise entitled. No study-specific interventions will be done before informed consent has been obtained.

The investigator will inform the participant of any relevant information that becomes available during the course of the study, and will discuss with them, whether they wish to continue with the study. If applicable they will be asked to sign revised consent forms.

If the Consent Form is amended during the study, the investigator shall follow all applicable regulatory requirements pertaining to approval of the amended Consent Form by the REC and use of the amended form (including for ongoing participants).

## RECORDS

### Study Forms

Each participant will be assigned a study identity code number, for use on study forms, other study documents and the electronic database. The documents and database will also use their initials (of first and last names separated by a hyphen or a middle name initial when available), date of birth (dd/mm/yy), date of scan and MRI volunteer code (as allocated by Mrs Lesley Martin at the SPMMRC). This 4 point identifier will reduce risk of errors linking participant information and MRI findings.

Study forms will be treated as confidential documents and held securely in accordance with regulations. The investigator will make a separate confidential record of the participant’s name, date of birth, local hospital number or NHS number, and Participant Study Number, to permit identification of all participants enrolled in the study, in case additional follow-up is required.

Study forms shall be restricted to those personnel approved by the Chief or local Investigator and recorded as such in the study records.’

All paper forms shall be filled in using black ballpoint pen. Errors shall be lined out but not obliterated by using correction fluid and the correction inserted, initialled and dated.

The Chief or local Investigator shall sign a declaration ensuring accuracy of data recorded in the study.

Monitoring of study data shall include confirmation of informed consent; source data verification; data storage and data transfer procedures; local quality control checks and procedures, back-up and disaster recovery of any local databases and validation of data manipulation. The Chief Investigator, or where required, a nominated designee, shall carry out monitoring of study data as an ongoing activity.

Data entries will be verified by inspection against the source data. A sample (10%) will be checked on a regular basis for verification of all entries made. In addition the subsequent capture of the data on any study databases will be checked. Where corrections are required these will carry a full audit trail and justification.

Study data and evidence of monitoring and systems audits will be made available for inspection by the regulatory authority as required.

### Source documents

Source documents shall be filed at the investigator’s site and may include but are not limited to, consent forms, study records and questionnaires, field notes and MRI images. A Study form may also completely serve as its own source data. Only study staff shall have access to study documentation other than the regulatory requirements listed below.

### Direct access to source data / documents

The study form and all source documents shall made be available at all times for review by the Chief Investigator, Sponsor’s designee and inspection by relevant regulatory authorities.

## *RECORD RETENTION AND ARCHIVING*

In compliance with the DH Research Governance Framework guidelines, the Human Tissue Act and in accordance with the University of Nottingham Research Code of Conduct, the Chief Investigator will maintain all records and documents of the study. These will be retained for at least 7 years or for longer if required. If the responsible investigator is no longer able to maintain the study records, a second person will be nominated to take over this responsibility.

The study documents held by the Chief Investigator on behalf of the Sponsor shall be finally archived at secure archive facilities at the University of Nottingham. This archive shall include all study databases and associated meta-data encryption codes.

## DATA PROTECTION

All study staff and investigators will endeavour to protect the rights of the study’s participants to privacy and informed consent, and will adhere to the Data Protection Act, 1998. The CRF will only collect the minimum required information for the purposes of the trial. CRFs will be held securely, in a locked room, or locked cupboard or cabinet. Access to the information will be limited to the trial staff and investigators and any relevant regulatory authorities (see above). Computer held data including the study database will be held securely and password protected. All data will be stored on a secure dedicated web server. Access will be restricted by user identifiers and passwords (encrypted using a one way encryption method).

Information about the study in the participant’s medical records / hospital notes will be treated confidentially in the same way as all other confidential medical information.

Electronic data will be backed up every 24 hours to both local and remote media in encrypted format.

# QUALITY ASSURANCE & AUDIT

## INSURANCE AND INDEMNITY

Insurance and indemnity for clinical study participants and study staff is covered within the NHS Indemnity Arrangements for clinical negligence claims in the NHS, issued under cover of HSG (96)48. There are no special compensation arrangements, but study participants may have recourse through the NHS complaints procedures.

The University of Nottingham has taken out an insurance policy to provide indemnity in the event of a successful litigious claim for proven non-negligent harm.

## STUDY CONDUCT

Study conduct will be subject to systems audit for inclusion of essential documents; permissions to conduct the study; CVs of study staff and training received; local document control procedures; consent procedures and recruitment logs; adherence to procedures defined in the protocol (e.g. inclusion / exclusion criteria, timeliness of visits); accountability of study materials and equipment calibration logs.

The Study Coordinator, or where required, a nominated designee of the Sponsor, shall carry out a site systems audit at least yearly and an audit report shall be made.

## STUDY DATA

Monitoring of study data shall include confirmation of informed consent; source data verification; data storage and data transfer procedures; local quality control checks and procedures, back-up and disaster recovery of any local databases and validation of data manipulation. The Study Coordinator, or where required, a nominated designee of the Sponsor, shall carry out monitoring of study data as an ongoing activity.

Entries on CRFs will be verified by inspection against the source data. A sample of CRFs (10%) will be checked on a regular basis for verification of all entries made. In addition the subsequent capture of the data on the study database will be checked. Where corrections are required these will carry a full audit trail and justification.

Study data and evidence of monitoring and systems audits will be made available for inspection by the REC as required.

## RECORD RETENTION AND ARCHIVING

In compliance with the ICH/GCP guidelines, regulations, the DH Research Governance Framework guidelines, the Human Tissue Act and in accordance with the University of Nottingham Research Code of Conduct, the Chief Investigator will maintain all records and documents of the study. These will be retained for at least 7 years or for longer if required. If the responsible investigator is no longer able to maintain the study records, a second person will be nominated to take over this responsibility.

The study documents held by the Chief Investigator on behalf of the Sponsor shall be finally archived at secure archive facilities at the University of Nottingham. This archive shall include all study databases and associated meta-data encryption codes.

## DISCONTINUATION OF THE TRIAL BY THE SPONSOR

The Sponsor reserves the right to discontinue this study at any time for failure to meet expected enrolment goals, for safety or any other administrative reasons. The Sponsor shall take advice as appropriate in making this decision.

## STATEMENT OF CONFIDENTIALITY

Individual participant medical or personal information obtained as a result of this study are considered confidential and disclosure to third parties is prohibited with the exceptions noted above.

Participant confidentiality will be further ensured by utilising identification code numbers to correspond to treatment data in the computer files.

Such medical information may be given to the participant’s medical team and all appropriate medical personnel responsible for the participant’s welfare.

Data generated as a result of this study will be available for inspection on request by the participating physicians, the University of Nottingham representatives, the REC, local R&D Departments, representatives of the Human Tissue Authority and the regulatory authorities.

# PUBLICATION AND DISSEMINATION POLICY

Results will be reported at scientific meetings and published in peer reviewed journals.

# USER AND PUBLIC INVOLVEMENT

There has been no public involvement in the design of this study.

# STUDY FINANCES

### Funding source

This study is funded by the Nottingham Digestive Disease Centre. Other funding sources are being sought.

### Participant stipends and payments

Participants will not be paid to participate in the study. Travel expenses will be offered for any hospital visits in excess of usual care up to a maximum of £50.

# SIGNATURE PAGES

Signatories to Protocol:

**Chief Investigator:** (name)__________________________________

Signature:__________________________________

Date: ___________

**Co- investigator**: (name) __________________________________

Signature:__________________________________

Date: ___________

**Study Statistician**: (name)__________________________________

Signature:__________________________________

Date: ___________

# REFERENCES
