# Supplementary material for: A pilot study of visceral fat and its association with adipokines, stool calprotectin and symptoms in patients with diverticulosis
Source: PLoS One. 2019 May 8;14(5):e0216528. doi: 10.1371/journal.pone.0216528 (PMC6505945; doi:10.1371/journal.pone.0216528)
Supplement: S1 Methods — (DOCX) [file pone.0216528.s003.docx]

**S1 Methods**

**Supporting Information**

1. **Materials**

Participants in the diverticular study were scanned at 1.5 T and we also included healthy volunteers who were scanned at both 1.5 T and 3.0 T as part of a separate repeatability study using a whole-body scanner (Achieva, Philips Medical System, The Netherlands). The repeatability study was reviewed and approved by University of Nottingham Medical School Ethics Committee (approval number H14082014/15). Volunteers were positioned supine in the scanner with a 16 element SENSE receive torso coil around the abdomen. The images to determine volumes of subcutaneous and visceral fat were acquired using a 2-echo 3D T1 weighted mDIXON protocol. The data were reconstructed to create in-phase, out-of-phase, water only and fat only images in the transverse plane. The data were acquired in 3 stacks; each stack (150 mm depth) acquired in a short breath hold (<20s). Parameters for both field strengths were matched to be comparable and were as follows: flip angle = 10^o^, SENSE = 2.0, TE_1_ = 1.8 ms, TE_2_ = 4 ms, TR = 5.4 ms, FOV 400-480 x 370-447 mm^2^ acquired resolution 1.5 x 1.95 x 6 mm^3^, reconstructed resolution 1.25 x 1.25 x 3 mm^3^, given 50 reconstructed slices in each stack. The algorithm was written using standard image processing tools available in IDL® 6.4 (Research Systems, Boulder Co, USA), including a graphical user interface.

1. **Procedure**
   1. **Description of Fat Segmentation Algorithm**

Following definition of the slices to be analysed, the next step was to define an Abdominal Mask which included all abdominal tissue whilst excluding any limbs, by applying a thresholded, region-growing technique to the data from all 4 image types. Any holes within this mask region were automatically filled in to define a continuous mask including the whole of the abdomen within the slab of interest (as indicated by the red outline on Fig 1A). The resulting Abdominal Mask was visually checked, so that the observer could recalculate the mask with a different threshold if necessary, for instance if the subject’s arms were close to body wall. These processes (loading data, selecting central slice and creating Abdominal Mask) took approximately 1 minute of operator time and 10s of computer time.


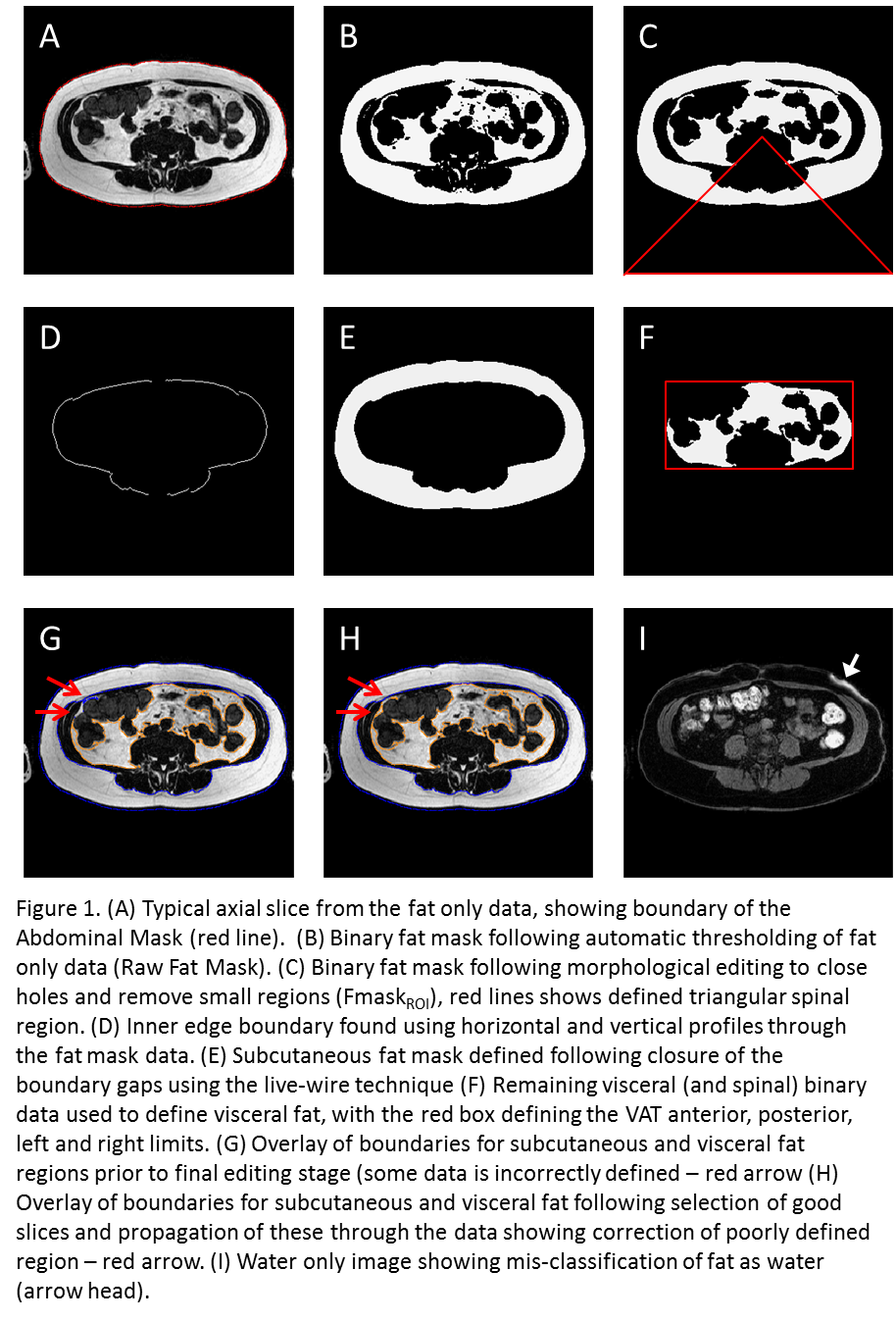


A histogram was created of the signal in the fat-only images from all voxels contained within the Abdominal Mask. The signal at which the minimum in the histogram plot between the fat peak and background tissue signal peak was chosen as a cut off and all pixels with signals greater than this were used to define a Raw Fat Mask [1] (Fig 1B). To reduce the effects of noise and blood vessels all holes smaller than 150 voxels within a single axial slice were automatically filled to produce an additional mask (Fmask_no_holes_) used for intermediate processing (size chosen to avoid filling gaps due to loops of small bowel or other organs). A 2D morphological eroding filter (3x3 kernel) was applied to all axial slices and subsequently any unconnected regions smaller than 100 voxels in a single slice were removed from this mask (100 voxels was chosen to remove fat deposits around the spine, which are general smaller than fat depots in the visceral region). This process was repeated in the coronal and sagittal directions and then a mask was then formed by combining the three orthogonal eroded datasets: if data remained in a voxel after erosion in at least 2 directions then the voxel was retained in the final combined mask (Fmask_erode_). The result of this process was sometime to lose protrusions of fat which were connected to the visceral fat in the Raw Fat Mask. Using the Fmask_no_holes_ axial data, regions were then ‘regrown’ from those which had remained in Fmask_erode_ provided that the ‘regrown’ data added at least 30 voxels to the region and that more than half of these were not in the spinal region defined as a triangular region (Fig 1C) with its apex at the centre of mass of the Abdominal Mask and its base at the back of the body [2]. This mask showed the regions of interest that contained the visceral and subcutaneous fat that were to be segmented and quantified in subsequent processing (Fmask_ROI_) (Fig 1C).

The next task was to separate the subcutaneous and visceral fat. To generate the subcutaneous fat mask (Fmask_SAT_) the inner boundary of the subcutaneous adipose tissue had to be defined. To achieve this, multiple 1D profiles were generated across the fat data in both the horizontal and vertical directions. The first point in each profile which was not in the mask after previously being in the mask (corresponding to adipose tissue), was defined as a boundary point. Boundary points which were connected to more than 19 other boundary points were retained into the boundary. Boundary points connected to more than 9 but less than 19 others were only retained if they were relatively close (< 10 voxels) to the external boundary of the Abdominal Mask. This allowed for a reduction in the number of gaps in the boundary, whilst not including data which was clearly not from the boundary region. This left gaps in the boundary definition (Fig 1D) which were automatically closed using a live-wire algorithm [3] based on a cost map which was weighted to the Raw Fat Mask and out-of-phase data (on which the boundary pixels of fat/water were dark). This boundary was then used to identify an initial estimate of the SAT Mask (Fig 1E).

The remaining pixels in the Fmask_roi_ contained visceral and potentially some subcutaneous fat (Fig 1F). Any small regions (< 500 pixels) that were connected to the subcutaneous fat region were automatically reassigned to the SAT Mask and removed from the VAT Mask. To define the outer limits of the visceral fat region all holes within this mask were automatically filled and it’s anterior, posterior, left and right boundaries calculated for each axial slice. Data from the Raw Fat Mask which had not been assigned to either the SAT mask or VAT region were then automatically categorised as either VAT, SAT or non-VAT (spinal and intra muscular) depending on the position of their regional centre of mass relative to the boundaries of the VAT region and Abdominal Mask centre of mass (see Table 1 for details). After assignment of all remaining regions in the Raw Fat Mask, the masks of VAT and SAT were used as the ‘initial guess’ for segmentation of the tissue. These processes (creating Raw Fat Mask, initial guess SAT and VAT masks) took on average 5 minutes of computer time, however this was increased if there were large gaps in the boundary of the SAT as the number of live wire calculations depend on the size of the gap to cross.

The observer viewed the boundaries of these masks (Fig 1G) to identify 3 - 8 anchor slices distributed throughout the dataset that they considered showed the correct separation of VAT and SAT. These anchor slices were then used to create the final masks by only allowing small variations (up to 2 voxels) of the boundary position between slices in the initial guess fat masks working out in both directions from each anchor slice, without allowing the data to be added in the spinal triangle (Fig 1H). Examples of this process are shown in Fig 2. These processes (defining anchor slices and creating final masks) took approximately 1 minute of operator time and 1 minute of computer time. Occasionally the algorithm above failed. This tended to happen if the VAT volume was very fragmented, or in subjects with large volumes of VAT where sacral fatty regions were included. Simple manual editing tools were provided to reclassify regions to be removed from the VAT region or by manually defining additional polygons to add extra ROIs to the VAT mask.

Volumes of VAT SAT and TAT adipose tissue were then calculated by applying the final VAT and SAT and Abdominal Masks to the Raw Fat Mask, and multiply the resulting number of voxels by the voxel size. Occasionally the mDIXON algorithm mislabelled fat as water (Fig 1I) so that this tissue was not included in the Raw Fat Mask but would have been included in the SAT mask above since the SAT mask was based on boundary definitions and not based directly on the signal intensity in the fat only image. To overcome this, a Water Mask was defined as all voxels in the top 20 % of the signal intensities from the water only images included (since the misclassified fat signals always have very high signal intensity). The Water Mask had a morphological opening filter applied to the data in each axial slice (3x3 kernel) to remove the skin signal and then regions larger than 10 voxels from this Water Mask, which were defined as TRUE on the SAT Mask but FALSE on the Raw Fat Mask, were included within the SAT total volume.

The final algorithm then output the VAT, SAT and TAT volumes and ratios of VAT/SAT and VAT/TAT.

- 1. **Validation Experiments**
     1. **Comparison to Manual Segmentation**

To determine whether the boundaries set for the VAT and SAT were correctly identified the semi-automated data was compared with manual segmentation. Fat only images from the 10 diverticular disease subjects with a wide range of BMIs were manually segmented into subcutaneous, spinal and visceral fat regions, by a single observer (MK) using the ‘smart edge’ tool in Analyze9™ (Mayo Foundation, Rochester, MN, USA). These were used in combination with the raw fat only images to generate masks of VAT, SAT and TAT using the same threshold to define the fat as for the semi-automatic method. A slice-by-slice analysis of the data was also carried out to show the variation in the measured volumes from the bottom to the top of the region examined.

- - 1. **Test-retest repeatability and cross-field experiments**

To determine the test-retest repeatability of the algorithm and compare data across two different field strengths (1.5 T and 3.0 T), 10 healthy volunteers were scanned on 2 separate days (one day at each field strength scanner). In each scanning session, the volunteers were scanned using the mDIXON sequence then removed from the scanner bed, repositioned back onto the scanner bed and rescanned using the same sequence. For the analysis of this data no additional editing was made to the data after the final automatic processing step. Subjects were not asked to fast before scanning, however they were scanned at similar times of the day at both field strengths.

Although 51 slices were used in the segmentation algorithm to allow adequate region growing, the final results from the repeatability were quoted for the central 30 slices where the algorithm was most stable, as shown by the manual segmentation results.

- - 1. **Inter-observer variability**

Two independent observers analysed the data from the diverticular disease patients to assess inter-observer variability.

1. **Results**

Comparison of the volumes from 10 diverticular disease patients measured using the semi-automatic algorithm and manual segmentation are given in Table 2.

Table 2. Comparison of manual and semi-automatic definitions of adipose tissue regions (N=10). 51 slice data shown at top of table and 30 slice data shown in shaded region below.

|  | **VOLUMES (ml)** | | | **RATIOS** | |
| --- | --- | --- | --- | --- | --- |
|  | **VAT** | **SAT** | **TAT** | **VAT/SAT** | **VAT/TAT** |
| Mean Semi-Auto Data | 1860±600 | 4020±1950 | 6050±2260 | 0.53±0.24 | 0.32±0.09 |
| Range | 1050-2310 | 1620-8540 | 3500-11070 | 0.23-1.0 | 0.18-0.46 |
| Mean Manual Data | 1860±610 | 3960±1920 | 6050±2260 | 0.54±0.23 | 0.32±0.09 |
| Range | 1030-2350 | 1590-8430 | 3500-11070 | 023-0.98 | 0.18-0.45 |
| B-A Bias (MAN-AUTO) | 1.6 | -50.5 | -0.3 | 0.005 | -0.001 |
| B-A 95%CI | -51.1-54.3 | -106.7-5.8 | -2.1-1.6 | -0.015-0.024 | -0.013-0.011 |
| Mean Dice Co-efficient | 0.982±0.011 | 0.992±0.002 | 1.000±0.000 | N/A | N/A |
| Mean Semi-Auto Data | 1020±310 | 2540±1180 | 3660±1350 | 0.46±0.19 | 0.29±0.08 |
| Range | 620-1490 | 1000-5230 | 2030-6690 | 0.20-0.85 | 0.16-0.42 |
| Mean Manual Data | 1020±320 | 2540±1170 | 3660±1350 | 0.46±0.19 | 0.29±0.08 |
| Range | 600-1500 | 1000-5230 | 2030-6690 | 0.20-0.86 | 0.16-0.42 |
| B-A Bias (MAN-AUTO) | -0.2 | -27.8 | 0.0 | 0.004 | -0.001 |
| B-A 95%CI | -25.2 to 24.8 | -62.4 to 6.8 | -0.2 to 0.2 | -0.008to 0.016 | -0.009 to 0.007 |
| Mean Dice Co-efficient | 0.983±0.009 | 0.994±0.002 | 1.000±0.000 | N/A | N/A |

A broad range of volumes were measured across the subjects for the different adipose tissue regions. The dice coefficients for all the different regions were greater than 0.95, showing excellent agreement between the boundaries defined by the algorithm and those defined by manual segmentation. Fig 2 shows how the differences between the manual and semi-automatic segmentation varied across the 51 slices.


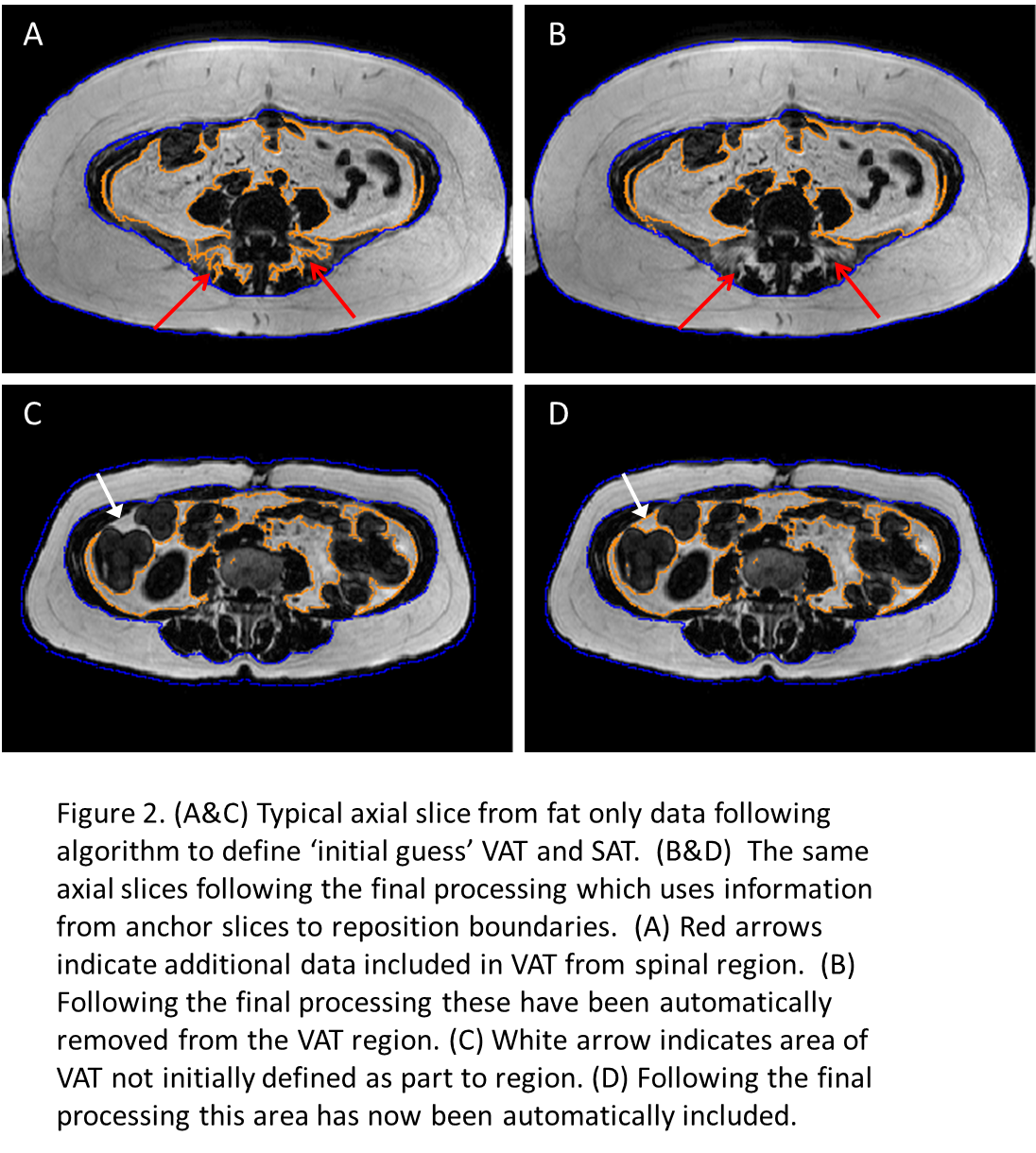


Although the mean differences between the two methods is low across all slices, the slices towards the top and bottom of the region had much larger standard deviations indicating a much wider spread in the differences. This was seen for the VAT in the pelvic region and SAT for the liver region. Fig 3 shows the variability of individual data across the 30 central slices and the differences between male and female subjects and highlights the rapidly changing ratios of VAT/SAT across the volume studied seen in some individuals. Male subjects had much greater changes of VAT volumes across the 30 slices compared to female subjects. For the majority of individuals, and for both male and female subjects, larger amounts of VAT were measured in the slices lower than the central slice compared to those above it.

Bland-Altman plots of the test-retest repeatability data are shown in Fig 4 and summarised in Table 3. There was again a wide range of volumes measured for the different adipose tissue regions, within the healthy volunteer group (VAT: 150 - 1040 mL, SAT: 1340 - 3170 mL, TAT: 1810 - 3770 mL, 3.0T data). There was very little bias between repeated measurements at the same field strength and between field strengths, with the bias representing just a few percent of the total volume measured. The limits of agreement were largest for the VAT relative to the volume size measured, however these limits were still small compared to the wide range of volumes measured across subjects. Limits for VAT were considerably wider for the 3T data compared to the 1.5T data; however the opposite was true for the SAT measurements, with the 1.5T data showing the largest limits. Intra-class correlation coefficients were high (>0.95, p<0.001) for all the parameters measured (VAT, SAT, TAT, and ratios) both for the test-retest data at the same field strength and for the data acquired at different field strengths. Coefficients of variance were below 10% for all measured parameters at both field strengths, with most less than 5%.

Inter-observer variability data is given in Table 3. Intra-class correlation coefficients for the two observers measurements of all adipose tissue volumes (VAT, SAT and TAT) and ratios (VAT/SAT, VAT/TAT) were high (>0.99, p<0.001). There was no bias between the observers and very small limits of agreement from the Bland-Altman analysis (Table 3). The coefficient of variance was lower for the observer study compared to the repeatability data.

**References**

1. Nakai R, Azuma T, Kishimoto T, Hirata T, Takizawa O, Hyon SH, et al. Development of a high-precision image-processing automatic measurement system for MRI visceral fat images acquired using a binomial RF-excitation pulse. Magnetic resonance imaging. 2010;28(4):520-6. Epub 2010/01/26. doi: 10.1016/j.mri.2009.12.019. PubMed PMID: 20096526.

2. Thormer G, Bertram HH, Garnov N, Peter V, Schutz T, Shang E, et al. Software for automated MRI-based quantification of abdominal fat and preliminary evaluation in morbidly obese patients. J Magn Reson Imaging. 2013;37(5):1144-50. Epub 2012/11/06. doi: 10.1002/jmri.23890. PubMed PMID: 23124651.

3. Barrett WA, Mortensen EN. Interactive live-wire boundary extraction. Med Image Anal. 1997;1(4):331-41. Epub 1999/01/05. doi: S1361-8415(97)85005-0 [pii]. PubMed PMID: 9873914.
